# Visual observation of photonic Floquet–Bloch oscillations

**DOI:** 10.1038/s41377-024-01419-z

**Published:** 2024-04-28

**Authors:** Zhen Zhang, Yuan Li, Xiankai Sun, Xuewen Shu

**Affiliations:** 1 Wuhan National Laboratory for Optoelectronics and School of Optical and Electronic Information, Huazhong University of Science and Technology, Wuhan, Hubei 430074 China; 2 Department of Electronic Engineering, The Chinese University of Hong Kong, Shatin, New Territories, Hong Kong SAR, China

**Keywords:** Optical physics, Optical materials and structures

## Abstract

Bloch oscillations (BOs), an important transport phenomenon, have been studied extensively in static systems but remain mysterious in Floquet systems. Here, by harnessing notions from photonic analogy, we propose a generalization of the existing BOs in photonic Floquet lattices, namely the “photonic Floquet–Bloch oscillations”, which refer to rescaled photonic Bloch oscillations with a period of extended least common multiple of the modulation period and the Bloch oscillation period. Next, we report the first visual observation of such photonic Floquet–Bloch oscillations (FBOs) by employing waveguide fluorescence microscopy. Most significantly, the FBOs surpass the existing BOs in Floquet systems and exhibit exotic properties on their own, including fractal spectrum and fractional Floquet tunneling. This new transport mechanism offers an intriguing method of wave manipulation that may contribute to rapidly developing fields in photonics, condensed matter physics, and quantum physics.

## Abstract (web summary)

We propose a general theory concerning Bloch oscillations in photonic Floquet lattices and report the first visual observation of the Bloch-like oscillations, which are termed the “photonic Floquet–Bloch oscillations.”

## Graphical abstract

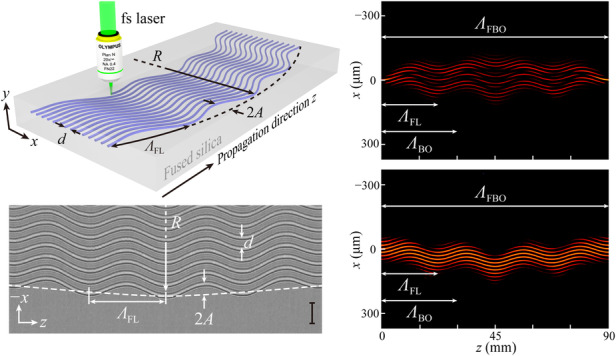

## Introduction

As a fundamental phenomenon of coherent quantum motion, Bloch oscillations (BOs), the oscillatory motion of a quantum particle with a BO period *Λ*_BO_, were first predicted by Bloch and Zener in the context of crystal under a constant electric field^1,2^. BOs were initially observed for matter waves within semiconductor superlattices^3^ and ultracold atoms^4,5^, revealing their nature as a wave phenomenon. Subsequently, BOs have been extended to various wave systems, including acoustic cavities^6,7^, waveguide arrays^8–12^, and synthetic frequency lattices^13–15^. In the past decade, abundant interesting phenomena concerning BOs were focused primarily on static systems^16–20^. Recently, the exploration of BOs in periodically driven quantum systems, equivalent as “Floquet systems”, has drawn tremendous attention because their exotic characteristics are profoundly distinct from those in static systems^21–37^. More specifically, two types of Bloch-like oscillations have been investigated as quasi-Bloch oscillations (QBOs)^21–23^ and super-Bloch oscillations (SBOs)^24–31^. QBOs occur with a period *Λ*_QBO_ = *Λ*_BO_ if the BO period *Λ*_BO_ is an integer multiple of the modulation period *Λ*_FL_, i.e., *Λ*_BO_ = *NΛ*_FL_ (*N* is a positive integer greater than 1). SBOs refer to rescaled BOs with super large oscillation amplitude and period, where the BO period *Λ*_BO_ (or its integer multiple) is slightly detuned from the modulation period *Λ*_FL_, i.e., *Λ*_FL_ ~ *NΛ*_BO_. Under similar schemes, these two phenomena seem to be intimately related. However, the underlying connection of these existing BOs in Floquet systems remains elusive, and a general theory concerning BOs in Floquet systems needs to be developed.

Furthermore, as a key to unraveling the mechanism of the underlying transport, the visual observation concerning BOs in Floquet systems is still experimentally challenging owing to the fast temporal evolution of the wavefunction in a quantum mechanical system. Recently, the concept of “photonic analogy” has emerged to address this challenge by simulating the temporal evolution of the wavefunction through the spatial light evolution in a waveguide array^38–42^. With the photonic analogy, the propagation coordinate *z* acts as “time” and the periodic drive implemented in waveguide trajectory gives rise to Floquet engineering^43–47^. Therefore, the photonic analogy has offered experimentally realistic configurations to verify various Floquet–Bloch theories.

In this article, we developed a general theory concerning BOs in photonic Floquet lattices and report the first visual observation of the photonic Bloch-like oscillations, which we called “photonic Floquet–Bloch oscillations (FBOs)”. The photonic FBOs refer to rescaled BOs with a motion period *Λ*_FBO_ of the extended least common multiple (LCM) of the Floquet modulation period *Λ*_FL_ and the BO period *Λ*_BO_. The photonic FBOs occur for arbitrary Floquet engineering when the rational ratio of *Λ*_FL_/*Λ*_BO_ is non-integer, i.e., *Λ*_FL_ ≠ *NΛ*_BO_. Under this framework, the conventional QBOs (*Λ*_BO_ = *NΛ*_FL_) and SBOs (*Λ*_FL_ ~ *NΛ*_BO_) can now be unified and treated as two special cases of FBOs (*Λ*_FL_ ≠ *NΛ*_BO_) with specific ratios *Λ*_FL_/*Λ*_BO_. Moreover, we directly visualized the breathing and oscillatory motions of photonic FBOs by employing waveguide fluorescence microscopy. In contrast to previous measurements that only recorded several profiles during one oscillation period^23–25^, the direct visualization reported here records the intricate details of continuum evolution.

Significantly, the visual observation contributes to revealing the key features of photonic FBOs. With this insight, we investigated two exotic properties of photonic FBOs, namely the fractal spectrum and fractional Floquet tunneling. Specifically, we found that the FBO period *Λ*_FBO_ is the Thomae’s function (a fractal spectrum) of the ratio *Λ*_BO_/*Λ*_FL_, and several peaks of such a fractal spectrum were experimentally confirmed. In addition, we experimentally demonstrated the Floquet-induced rescaling of the FBO amplitude with a varying amplitude *A* of harmonic modulation, which refers to fractional Floquet tunneling. Beyond the conventional tunneling that follows an integral-order Bessel function *B*_*v*_(*A*)^35–40^, such fractional Floquet tunneling of FBO amplitude follows a linear combination of fractional-order Anger *J*_*v*_(*A*) and Weber functions *E*_*v*_(*A*). Hence, photonic FBOs constitute a unique transport phenomenon on their own, in addition to being a generalization of the existing BOs in Floquet systems.

## Results

### Theory of BOs in a photonic Floquet lattice

**Fig. 1:**
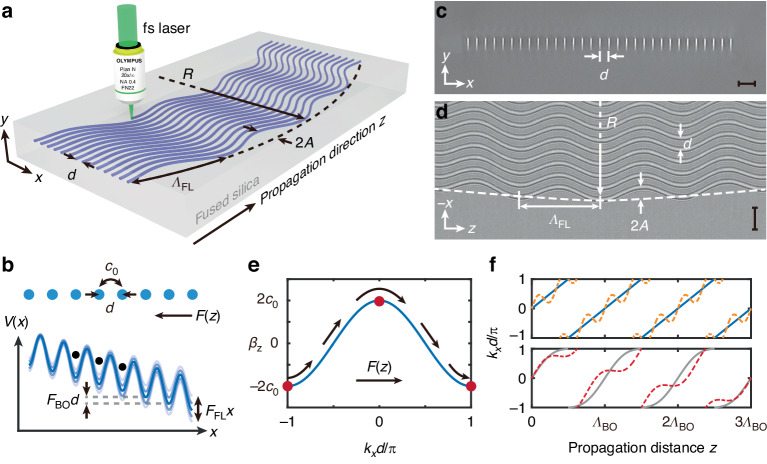
**Photonic implementation and generalized acceleration theory.** **a** Schematic of a one-dimensional lattice composed of evanescently coupled waveguides with combined bending trajectory. **b** Schematic of a reduced Floquet lattice in the transformed coordinate frame. **c** Cross-sectional optical microscope image of the fabricated sample. Scale bar, 30 μm. **d** Top-view optical microscope image of the fabricated sample with a harmonic modulation. Scale bar, 30 μm. **e** Representation of *F*(*z*)-induced wave vector shift according to the generalized acceleration theory. **f** *z*-dependent shift of the transverse Bloch momentum for several specific cases corresponding to conventional BOs (*A* = 0, blue solid line), FBOs (*Λ*_BO_ = 3*Λ*_FL_, orange dashed line), FBOs (3*Λ*_BO_ = 4*Λ*_FL_, red dashed line), and spreading (*Λ*_BO_ = *Λ*_FL_, gray solid line)

Here, we employ a femtosecond-laser-written waveguide array^48–51^ in a fused silica substrate (Corning 7980) as an experimental platform for visualizing BOs in a photonic Floquet lattice. As depicted in Fig. 1a, we first considered a curved photonic lattice that consists of identical waveguides with waveguide spacing *d* and array length *L*. In the transverse direction *x*, the center of each waveguide core varies along the longitudinal direction *z* by following a combined trajectory according to *x*_0_(*z*) = *x*_BO_(*z*) + *x*_FL_(*z*), where *x*_BO_(*z*) = [*R*^2^ − (*z* – *L*/2)^2^]^1/2^ is the circular bending term with a bend radius *R*, and *x*_FL_(*z*) = *M*(*z*) is the periodic bending term with a modulation period *Λ*_FL_ and modulation function *M*(*z*) that satisfies *M*(*z*) = *M*(*z* + *Λ*_FL_). In the case of paraxial propagation along the longitudinal direction *z*, the envelope *ψ*(*x*, *y*, *z*) of the optical field guided in this photonic lattice at operating wavelength *λ* is governed by the Schrödinger-type equation:

1 $$i\frac{\partial \psi }{\partial z}=-\frac{1}{2{k}_{0}}{\nabla }^{2}\psi -\frac{{k}_{0}\Delta n(x,y,z)}{{n}_{0}}\psi$$

where ${\nabla }^{2}={\partial }_{x}^{2}+{\partial }_{y}^{2}$ is the Laplacian operator in the transverse plane, *n*_0_ ~ 1.46 is the refractive index of the substrate, *k*_0_ = 2π*n*_0_/*λ* is the wave number, and Δ*n*(*x*, *y*, *z*) = *n*(*x*, *y*, *z*) – *n*_0_ is the femtosecond-laser-induced refractive-index increase (Δ*n* > 0) that defines the entire photonic lattice. By considering a reference coordinate frame where the waveguides are straight in the $\tilde{z}$ direction, namely: $\tilde{x}=x+{x}_{0}(z)$, $\tilde{y}=y$, and $\tilde{z}=z$, the paraxial equation in the transformed coordinates can be expressed as

2 $$i\frac{\partial \tilde{\psi }}{\partial \tilde{z}}=-\frac{1}{2{k}_{0}}{\tilde{\nabla }}^{2}\tilde{\psi }-\frac{{{{k}}}_{0}}{{{{n}}}_{0}}[\Delta {{n}}(\tilde{{{x}}},\tilde{{{y}}},\tilde{{{z}}})+{{F}}(\tilde{{{z}}})\tilde{{{x}}}]\tilde{\psi }$$

with $\tilde{\psi }=\psi (\tilde{x},\tilde{y},\tilde{z})\exp \left\{-\frac{{ik}_{0}}{2{\rm{\pi }}}{\partial }_{\tilde{z}}x_{0}(\tilde{z})\tilde{x}-\frac{{ik}_{0}}{4{\rm{\pi }}}{\int }_{0}^{\tilde{z}}{[{\partial }_{\tilde{z}}x_{0}(\tau )]}^{2}d\tau \right\}$ and $F(\tilde{z})=-n_{0}{\partial }_{\tilde{z}}^{2}x_{0}(\tilde{z})$. The additional term $F(\tilde{z})$ is determined by the combined trajectory and can be separated into two terms, i.e., $F(\tilde{z})={F}_{\text{BO}}+{F}_{\text{FL}},$ with *F*_BO_ ~ *n*_0_/*R* (with *R* significantly larger than *L*) and ${F}_{\text{FL}}=-n_{0}{\partial }_{\tilde{z}}^{2}M(\tilde{z})$.

By using the notions from the photonic analogy, Eq. (2) indicates that the spatial evolution of low-power light in the proposed lattice is analogous to the temporal evolution of noninteracting electrons in a periodic potential subject to an electric field. As sketched in Fig. 1b, the spatial coordinate $\tilde{z}$ acts as “time” *t*, the periodic bending trajectory of each waveguide *x*_FL_(*z*) records the “time”-dependent information, the term ${F}_{\text{FL}}(\tilde{z})\tilde{x}$ serves as the Floquet engineering, and the effective potential $[\Delta n(\tilde{x},\tilde{y},\tilde{z})+{F}_{\text{BO}}\tilde{x}]$ refers to a sign-reversed linearly tilted potential $-V(\tilde{x})$ that gives rise to photonic BOs. Therefore, our proposed scheme provides an experimental realization of BOs in a photonic Floquet lattice.

Figure 1c displays the cross-sectional microscope image of a fabricated sample. Each waveguide in our sample supports a well-confined fundamental mode, allowing the application of nearest-neighbour tight-binding approximation, so the propagation of guided light can be described by the following set of coupled equations:

3 $$i\frac{\partial {a}_{m}}{\partial z}={-c}_{0}({a}_{m-1}+{a}_{m+1})-\frac{{k}_{0}{mF}(z)d}{{n}_{0}}{a}_{m}$$

where *a*_*m*_ is the amplitude of the guided mode ${|m}\rangle$ in the *m*th waveguide and *c*_0_ is the coupling constant between the nearest-neighbour waveguides. In the absence of force *F*(*z*), i.e., for straight waveguide arrays, introducing a plane wave ansatz *a*_*m*_ ∝ exp[*i*(*β*_*z*_*z − mk*_*x*_*d*)] into Eq. (3) yields the single-band dispersion *β*_*z*_(*k*_*x*_) = 2*c*_0_cos(*k*_*x*_*d*) (blue line in Fig. 1e), where *β*_*z*_(*k*_*x*_) denotes the longitudinal propagation constant and *k*_*x*_ denotes the transverse Bloch momentum. According to the generalized acceleration theory^27^, the presence of force *F*(*z*) leads to a shift of the transverse Bloch momentum ${k}_{x}(z)={k}_{x}(0)+\frac{{k}_{0}}{{n}_{0}}{\int }_{0}^{z}F(\tau )d\tau$ and the Houston function ${|{\psi }_{m,{k}_{x}}(z){{\rangle }}}=\exp \left\{-\frac{{{ik}}_{0}}{{n}_{0}}{\int }_{0}^{z}{\beta }_{z}[{k}_{x}(\tau )]d\tau \right\}|{\psi }_{m,{k}_{x}(z)}{{\rangle }}$ is the reconstructed solution (Supplementary Note 1). When *PΛ*_BO_ = *QΛ*_FL_ (*Q*, *P* are mutually prime integers), the extended LCM of *Λ*_BO_ and *Λ*_FL_ is defined as LCM(*Λ*_BO_, *Λ*_FL_) = *PΛ*_BO_ = *QΛ*_FL_, and *β*_*z*_[*k*_*x*_(*z*)] is a *z*-periodic function with a period *Λ*_FBO_ = LCM(*Λ*_FL_, *Λ*_BO_) (Supplementary Note 1). Consequently, the integral of *β*_*z*_[*k*_*x*_(*z*)] can be expressed as a sum of a linear function and a periodic function, i.e., ${\int }_{0}^{z}{\beta }_{z}[{k}_{x}(\tau )]d\tau =\varepsilon ({k}_{x})z+P(z)$ with *P*(*z*) = *P*(*z* + *Λ*_FBO_). As a result, the entire lattice be mapped onto another Floquet lattice, since the Houston function can be reduced to Floquet states as

4 $$|{\psi }_{m,{k}_{x}}(z){{\rangle }}=\exp \left[-\frac{{iz}}{{k}_{0}}\varepsilon ({k}_{x})\right]|{u}_{m,{k}_{x}(z)}{{\rangle }}$$

where $|{u}_{m,{k}_{x}(z)}{{\rangle }}=\exp \left\{-\frac{i}{{k}_{0}}{\int }_{0}^{z}{\beta }_{z}[{k}_{x}(\tau )]-\varepsilon ({k}_{x})d\tau \right\}|{\psi }_{m,{k}_{x}(z)}{{\rangle }}=|{u}_{m,{k}_{x}(z+{\varLambda }_{\text{FBO}})}{{\rangle }}$ is known as the Floquet function and $\varepsilon ({k}_{x})\equiv \frac{1}{{\varLambda }_{\text{FBO}}}{\int }_{0}^{{\varLambda }_{\text{FBO}}}{\beta }_{z}[{k}_{x}(\tau )]d\tau$ is the corresponding Floquet dispersion that provides the effective transport properties over a period *Λ*_FBO_. Under the single-band approximation, the Floquet dispersion is expressed as

5 $$\varepsilon ({k}_{x})=\mathop{\sum}\limits_{n=1}^{{{\varLambda }}_{{\rm{FBO}}}/{{\varLambda }}_{{\rm{FL}}}}\cos \left(\frac{2\uppi {{\varLambda }}_{{\rm{FL}}}}{{{\varLambda }}_{{\rm{BO}}}}n\right)D({k}_{x})$$

where $D({k}_{x})=\frac{{2c}_{0}}{{\varLambda }_{\text{FBO}}}{\int }_{0}^{{\varLambda }_{\text{FL}}}\cos \left[{k}_{x}(0)d-\frac{2{\rm{\pi }}\tau }{{\varLambda }_{\text{BO}}}-{k}_{0}{d\left.{\partial }_{z}M(z)\right|}_{0}^{-\tau }\right]d\tau$ in general contributes nonflat dispersion. Equation (5) implies that there are two possibilities for BOs in a photonic Floquet lattice. When *Λ*_FL_ ≠ *NΛ*_BO_, a complete cancellation of all orders of diffraction $\mathop{\sum }\nolimits_{n=1}^{{\varLambda }_{\text{FBO}}/{\varLambda }_{\text{FL}}}\cos \left(\frac{2{{{\uppi} }}{\varLambda }_{\text{FL}}}{{\varLambda }_{\text{BO}}}n\right)=0$ results in flat Floquet dispersion *ε*(*k*_*x*_) ≡ 0, indicating that the state experiences a periodic motion and returns to the initial state after propagating a period *Λ*_FBO_. We call this phenomenon “Floquet–Bloch oscillations”, because it is a combined phenomenon of Floquet engineering and Bloch oscillations. When *Λ*_FL_ = *NΛ*_BO_, the Floquet dispersion $\varepsilon ({k}_{x})\equiv \frac{{2c}_{0}}{{\varLambda }_{\text{FL}}}{\int }_{0}^{{\varLambda }_{\text{FL}}}\cos \left[{k}_{x}(0)d-\frac{2{\rm{\pi }}\tau }{{\varLambda }_{\text{BO}}}-{k}_{0}{d\left.{\partial }_{z}M(z)\right|}_{0}^{-\tau }\right]d\tau$ is in general no longer flat and the state experiences spreading. We emphasize that the above conclusions are valid for an arbitrary modulation function *M*(*z*). In this connection, the existing BOs under specific modulation, namely QBOs (*Λ*_BO_ = *NΛ*_FL_) and SBOs (*Λ*_FL_ ~ *NΛ*_BO_), can be unified and treated as two special cases of FBOs (*Λ*_FL_ ≠ *NΛ*_BO_) with specific ratios *Λ*_FL_/*Λ*_BO_.

### Visual observation of BOs in photonic Floquet lattices

To illustrate the similarity and difference between FBOs and the existing BOs in Floquet systems, we employed a harmonic modulation *M*(*z*) = *A*cos(2π*z*/*Λ*_FL_) (see Fig. 1d), where *A* denotes modulation amplitude. Without loss of generality, we considered four specific scenarios that correspond to conventional BOs (*A* = 0), FBOs (*Λ*_BO_/*Λ*_FL_ = 3), FBOs (*Λ*_BO_/*Λ*_FL_ = 4/3), and spreading (*Λ*_BO_/*Λ*_FL_ = 1). The corresponding shifts of the transverse Bloch momentum according to the generalized acceleration theory: ${k}_{x}(z)={k}_{x}(0)+\left[\frac{2{{\uppi}} z}{{\varLambda }_{\text{BO}}d}+\frac{2{{\uppi}} A{k}_{0}}{{\varLambda }_{\text{FL}}}\sin \left(\frac{2{{\uppi}} z}{{\varLambda }_{\text{FL}}}\right)\right]$ are displayed in Fig. 1f, where the harmonic modulation contributes a sub-oscillation to the states with Bloch-momentum-oscillation amplitude (2π*Ak*_0_)/*Λ*_FL_. In the latter three scenarios, we considered the modulation amplitude *A* = *A*_0_*Λ*_FL_/*Λ*_BO_ so that the sub-oscillation amplitude was normalized to (2π*A*_0_*k*_0_)/*Λ*_BO_.

To experimentally verify our prediction, we fabricated a set of 90-mm-long samples composed of 31 identical waveguides with a waveguide spacing *d* = 16 μm. With such a waveguide spacing *d*, the coupling coefficient between straight waveguides *c*_0_ ~ 1.45 cm^−1^ was experimentally characterized. These waveguides follow the combined trajectories having a bend radius *R* = 110.8 cm (corresponding to *Λ*_BO_ ~ 30 mm) and the modulation period *Λ*_FL_ = 10, 22.5, and 30 mm (corresponding to the ratios *Λ*_BO_/*Λ*_FL_ = 3, 4/3, and 1, respectively). With the considered modulation period, *A*_0_ = 18 μm was chosen to reduce the associated radiation losses of waveguides.

Similar to the existing BOs, FBOs exhibit a breathing and an oscillatory motion under a single-site excitation and a broad-beam excitation, respectively. In the following experiments, we implemented visible-light excitation (*λ* = 633 nm) and directly visualized both the breathing modes and oscillating modes of FBOs by using waveguide fluorescence microscopy^39,52^. As the key features of BOs in Floquet lattice, the sub-oscillations are clearly presented here, which have not been experimentally observed before. A coordinate transformation that maps circular arcs into straight lines was applied to digitally process the fluorescence image so that the light evolution could be visualized more intuitively. Further details of the sample fabrication and fluorescence imaging characterization are provided in Supplementary Note 2,3.

First, we focus on the breathing modes under a single-site excitation. The narrow excitation in the real space corresponds to a broad excitation of Bloch modes in the reciprocal space, resulting in strongly diffracting wave packets. To quantify the diffraction of wave packets for the single-site excitation, we define the variance of excitation at the distance *z* in such a discrete system as

6 $${\sigma }^{2}(z)=\frac{{\sum }_{m}{{m}^{2}\text{|}{\it a}_{\it m}\text{|}}^{2}}{{\sum }_{m}{\text{|}{\it a}_{\it m}\text{|}}^{2}}$$

**Fig. 2:**
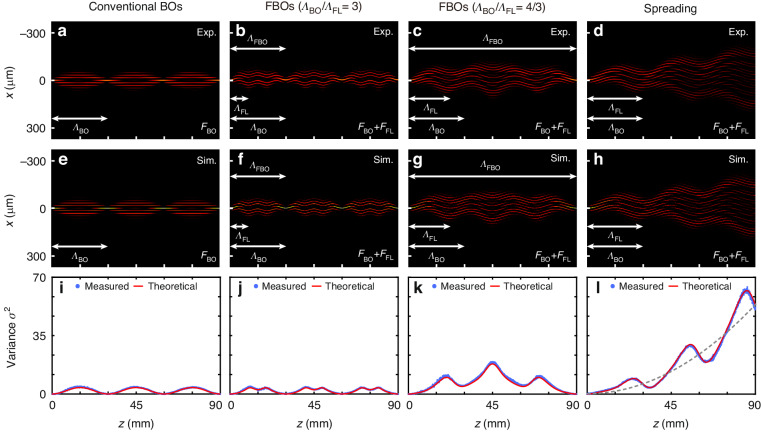
**Experimental visualization, simulation, and variance of the breathing modes for single-site excitation.** **a**–**d** Fluorescence microscopy images of the wave evolution in curved waveguide arrays with a fixed circular bend radius *R* = 110.8 cm (corresponding to *Λ*_BO_ = 30 mm). **a** *A* = 0, corresponding to conventional BOs; **b** *A* = 6 μm and *Λ*_FL_ = 10 mm, corresponding to QBOs; **c** *A* = 13.5 μm and *Λ*_FL_ = 22.5 mm, corresponding to SBO-like oscillations; **d** *A* = 18 μm and *Λ*_FL_ = 30 mm, corresponding to spreading. **e**–**h** Simulated wave evolution corresponding to those in **a**–**d**. **i**–**l** Corresponding variances *σ*^2^ of the measured and simulated light evolution as a function of the propagation distance *z*

The light is initially excited in the central waveguide resulting in a vanishing variance *σ*^2^(0) = 0, and a rise of the variance indicates that the light experiences broadening. Under the single-site excitation, the experimental results, respective simulations, and extracted variances *σ*^2^(*z*) for the scenarios considered in Fig. 1f are summarized in Fig. 2, where the first, second, third, and fourth columns correspond to conventional BOs (*A* = 0), FBOs (*Λ*_BO_/*Λ*_FL_ = 3), FBOs (*Λ*_BO_/*Λ*_FL_ = 4/3), and spreading (*Λ*_BO_/*Λ*_FL_ = 1), respectively. Without modulation (*A* = 0), Fig. 2a, e displays the light evolution that corresponds to conventional BOs, where the measured BO period ~30 mm is consistent with its theoretical value *Λ*_BO_ = *Rλ*/(*n*_0_*d*). The light first broadens until it propagates half of the BO period and then focuses into the central waveguide again at the BO period, as *σ*^2^ reaches its maximum at *z* ~ 15 mm and then decreases to zero at *z* ~ 30 mm (see Fig. 2i). When the modulation is introduced, BOs in the Floquet lattice exhibit diverse transport properties as expected, where the ratio *Λ*_BO_/*Λ*_FL_ makes a significant difference. For *Λ*_BO_/*Λ*_FL_ = 3, the FBOs are observed and degenerate into conventional QBOs, where the FBO period *Λ*_FBO_ is equal to the BO period *Λ*_BO_ (see Fig. 2b, f). The QBOs pattern is basically similar to that of conventional BOs, except that light experiences additional sub-oscillations, as *σ*^2^ oscillates with dual periods (see Fig. 2j). For *Λ*_BO_/*Λ*_FL_ = 4/3, the FBOs exhibit their similarity to SBOs, where the FBO period *Λ*_FBO_ ~ 90 mm is much longer than the BO period *Λ*_BO_ (see Fig. 2c, g). In addition to the extended FBO period, we also observed dramatic broadening of the light, as the maximum of *σ*^2^ is far larger than that of conventional BOs (see Fig. 2k). For *Λ*_BO_/*Λ*_FL_ = 1, the evolution of light propagating from 0 to *Λ*_FBO_/2 cannot be canceled with that propagating from *Λ*_FBO_/2 to *Λ*_FBO_. As a result, photonic FBOs are destroyed and spreading occurs, where light exhibits ballistic spreading and is no longer localized (see Fig. 2d, h). The discrete diffraction pattern accompanied by oscillations is observed, as *σ*^2^ oscillates around the gray-dashed curve of $2{c}_{0}^{2}{B}_{1}^{2}\left(\frac{2{\rm{\pi }}Ad{k}_{0}}{{\varLambda }_{\text{FL}}}\right){z}^{2}$ where *B*_1_ is the first-order Bessel function (see Fig. 2l).

Next, we focus on the oscillation modes under a broad-beam excitation. The broad-beam excitation in the real space corresponds to a narrow excitation in the reciprocal space. In this case, the group velocity of beam motion in the lattices can be expressed as *V*_group_(*z*) = −*dβ*_*z*_(*z*)/*dk*_*x*_(*z*) = 2*dc*_0_sin[*k*_*x*_(*z*)*d*], and the transverse displacement Δ*x*(*z*) of beam center is determined by $\Delta x(z)={\int }_{0}^{z}{V}_{\text{group}}(\tau )d\tau$. Here we define the weighted average position of excitation at the distance *z* in such a discrete system as

7 $$x(z)=\frac{{\sum }_{m}{{md}\text{|}{\it a}_{\it m}\text{|}}^{2}}{{\sum }_{m}{\text{|}{\it a}_{\it m}\text{|}}^{2}}$$

**Fig. 3:**
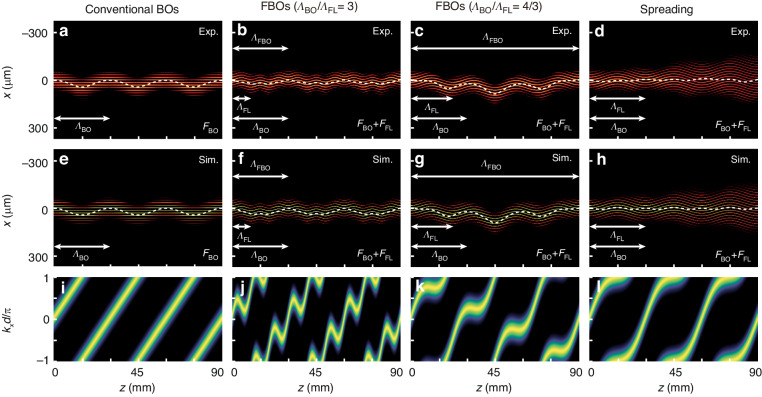
**Experimental visualization and simulation of the oscillating modes for broad-beam excitation.** **a**–**d** Fluorescence microscopy images of the wave evolution in the curved waveguide arrays with a fixed circular bend radius *R* = 110.8 cm (corresponding to *Λ*_BO_ = 30 mm). **a** *A* = 0, corresponding to conventional BOs; **b** *A* = 6 μm and *Λ*_FL_ = 10 mm, corresponding to QBOs; **c** *A* = 13.5 μm and *Λ*_FL_ = 22.5 mm, corresponding to SBO-like oscillations; **d** *A* = 18 μm and *Λ*_FL_ = 30 mm, corresponding to spreading. **e**–**h** Simulated wave evolution corresponding to those in (**a**–**d**). The trajectories of the beam *x*(*z*) extracted from the measured (**a**–**d**) and simulated (**e**–**h**) light evolution are marked as white dashed lines. **i**–**l** Corresponding simulated *z*-dependent shift of the transverse Bloch momentum obtained by projecting the wave evolution into the reciprocal space with spatial Fourier transform *W*(*k*_*x*_) = ∫*W*(*r*_*x*_)exp(−*jk*_*x*_⋅*r*_*x*_)*dr*

The excitation is located at the center of the lattice, i.e., *x*(0) = 0. During propagation, a rise (drop) of *x*(*z*) indicates that the light shifts toward the *x* (−*x*) direction. Here, we launched a 7-waveguide-wide Gaussian beam at normal incidence to the edge of the substrate. This corresponds to a narrow spectrum centered at *k*_*x*_(0) = 0 in the reciprocal space. Under the broad excitation, the experimental results, respective simulations, extracted trajectories of the beam *x*(*z*) (white dashed lines), and simulated acceleration of transverse Bloch momentum for the scenarios considered in Fig. 1f are summarized in Fig. 3, where the first, second, third, and fourth columns correspond to conventional BOs (*A* = 0), FBOs (*Λ*_BO_/*Λ*_FL_ = 3), FBOs (*Λ*_BO_/*Λ*_FL_ = 4/3), and spreading (*Λ*_BO_/*Λ*_FL_ = 1), respectively. Without modulation (*A* = 0), Fig. 3a, e display the light evolution that corresponds to conventional BOs, where the broad beam undergoes a sinusoidal oscillation with the BO period *Λ*_BO_. Similar to the breathing motion discussed previously, the oscillating motion exhibits diverse transport properties when the modulation is introduced. For *Λ*_BO_/*Λ*_FL_ = 3, Fig. 3b, f display the light evolution of FBOs that degenerate into conventional QBOs, where the trajectory of the broad beam following a sub-oscillating function was observed. The broad beam evolves along the *x* direction and returns to the initial position after propagating any multiple of the BO period *Λ*_BO_ ~ 30 mm. For *Λ*_BO_/*Λ*_FL_ = 4/3, Fig. 3c, g display the light evolution of FBOs that exhibit their similarity to SBOs, where the trajectory of the broad beam follows a giant sub-oscillating function with an extended period of ~90 mm. The maximal displacement of the broad beam for these SBOs-like oscillations is observed at half of the FBO period, i.e., *z* ~ 45 mm. For *Λ*_BO_/*Λ*_FL_ = 1, Fig. 3d, h display the light evolution that corresponds to spreading. Although the trajectory of the broad beam follows an oscillating function, beam broadening is observed during propagation. As a result, the beam does not return to the initial state of excitation, and photonic FBOs are destroyed.

For both single-site and broad-beam excitations, the visual observations of fluorescence images and quantitative analyses have excellent agreement with the respective simulation results. Therefore, our waveguide arrays are capable of accurately revealing BOs in photonic Floquet lattices.

### Fractal spectrum and fractional Floquet tunneling

In this section, we further provided quantitative analysis and investigated two exotic properties of photonic FBOs, namely fractal spectrum and fractional Floquet tunneling. These properties of photonic FBOs not only clarify their profound connection to the existing BOs in Floquet systems, but also reveal the way photonic FBOs constitute a unique phenomenon on their own. The detailed theoretical derivations and experimental results are provided in Supplementary Note 5, 6.

**Fig. 4:**
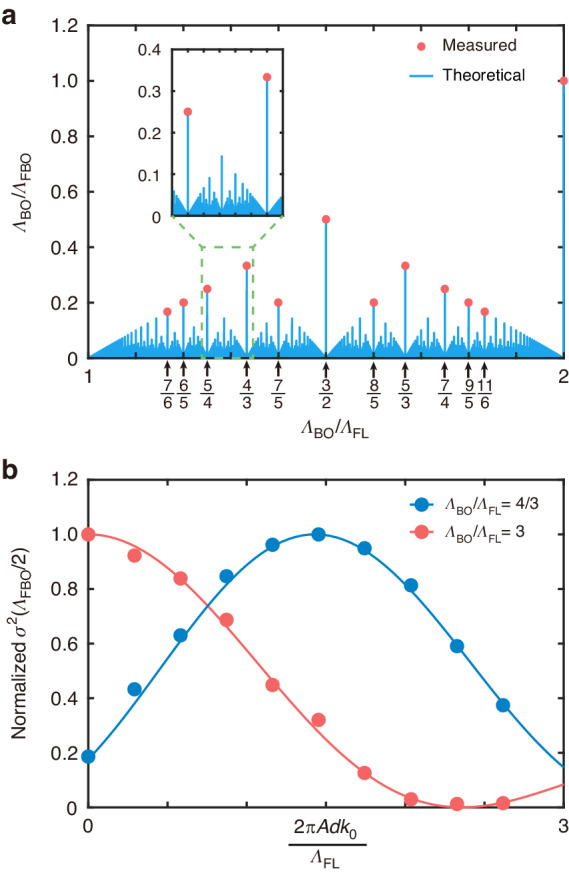
**Fractal spectrum and fractional Floquet tunneling of FBOs.** **a** Theoretical (blue stems) and measured (red dots) ratio *Λ*_BO_/*Λ*_FBO_ as a function of the ratio *Λ*_BO_/*Λ*_FL_. The inset is a close-up spectrum at a finer scale, which shows the property of self-similarity of this spectrum. **b** Normalized theoretical (lines) and measured (dots) FBO amplitude *σ*^2^(*Λ*_FBO_/2) as a function of the ratio *A*/*Λ*_FL_

Firstly, we studied the dependence of *Λ*_BO_/*Λ*_FBO_ on *Λ*_BO_/*Λ*_FL_ and investigated the fractal spectrum. As shown in Fig. 4a, the theoretically predicted FBO period *Λ*_FBO_ = LCM(*Λ*_BO_, *Λ*_FL_) determines that the FBO period spectrum follows the Thomae’s function when *Λ*_BO_/*Λ*_FL_ belongs to (1, 2). One may find that Thomae’s function is a fractal structure composed of infinite discrete peaks, where the patterns exhibit self-similarity at increasingly smaller scales^53^. Owing to limited sample lengths, we fabricated a set of samples with *Λ*_BO_/*Λ*_FBO_ ≥ 1/6, fixed *Λ*_BO_ = 30 mm, and varied *Λ*_FL_ from 15 to 30 mm. As expected, we experimentally verified several peaks of such a fractal spectrum by fitting the measured and simulated variance *σ*^2^(*z*) under single-site excitation. This fractal spectrum clarifies the profound connection between the existing BOs in Floquet systems and FBOs. The Thomae’s function can be approximated to a continuous linear function for a small detuning limit (*Λ*_BO_/*Λ*_FL_ approaches 1), indicating that the FBOs degenerate into conventional SBOs with a period given by *Λ*_SBO_ = *Λ*_FL_*Λ*_BO_/(*Λ*_BO_ − *Λ*_FL_). When *Λ*_BO_/*Λ*_FL_ equals to 1, the light experiences spreading as the FBO period approaches infinity. The situation is no longer the same when *Λ*_BO_/*Λ*_FL_ equals 2, where the FBOs degenerate into conventional QBOs with a period *Λ*_QBO_ = *Λ*_BO_. Most importantly, the existence of FBOs is experimentally confirmed for fractional *Λ*_BO_/*Λ*_FL_ (marked by the black arrows in Fig. 4a), which goes far beyond the existing BOs in Floquet systems. These peaks are the epitome of the entire spectrum that reveals the fractal nature of the FBOs: their period *Λ*_FBO_ has complex and seemingly random dependence on *Λ*_BO_/*Λ*_FL_.

Secondly, we studied the dependence of FBO amplitude [defined as *σ*^2^(*Λ*_FBO_/2)] on modulation amplitude *A* and investigated the fractional Floquet tunneling. Under the single-site excitation, the introduction of harmonic modulation leads to a rescaling of FBO amplitude following the square of a linear combination of the Anger function ${J}_{v}\left(\frac{2{\rm{\pi }}Ad{k}_{0}}{{\varLambda }_{\text{FL}}}\right)$ and the Weber function ${E}_{v}\left(\frac{2{\rm{\pi }}Ad{k}_{0}}{{\varLambda }_{\text{FL}}}\right)$ with a fractional-order *v* = *Λ*_FL_/*Λ*_BO_. Figure 4b displays two examples of such fractional Floquet tunneling, including QBOs (red line, *Λ*_BO_/*Λ*_FL_ = 3) and SBOs-like oscillations (blue line, *Λ*_BO_/*Λ*_FL_ = 4/3). Each curve is normalized to unity at its maximum. For the QBOs, the theoretically predicted FBO amplitude has a characteristic ${\left[2\cos ({\rm{\pi }}/3){E}_{1/3}\left(-\frac{2{\rm{\pi }}Ad{k}_{0}}{{\varLambda }_{\text{FL}}}\right)+2\sin ({\rm{\pi }}/3){J}_{1/3}\left(-\frac{2{\rm{\pi }}Ad{k}_{0}}{{\varLambda }_{\text{FL}}}\right)\right]}^{2}$ dependence on *A*/*Λ*_FL_. By contrast, the Floquet tunneling for the SBOs-like oscillations exhibits a different behavior, where the FBO amplitude has a characteristic $8{{J}_{3/4}\left(\frac{2{\rm{\pi }}Ad{k}_{0}}{{\varLambda }_{\text{FL}}}\right)}^{2}$ dependence on *A*/*Λ*_FL_. To verify our prediction, we fabricated two sets of samples with a varied modulation amplitude *A* and extracted the corresponding variance *σ*^2^(*z*) from the measured fluorescence images. For the QBOs, with increasing amplitude *A,* the FBO amplitude decreases before it reaches zero, indicating that the introduction of harmonic modulation will not broaden the FBO amplitude compared with the conventional BOs (*A* = 0). For the SBOs-like oscillations, with increasing amplitude *A* the FBO amplitude first increases to its maximum around *A* = 22.5 μm and then decreases. We emphasize that the proposed fractional Floquet tunneling provides a flexible way to manipulate the light that goes beyond the conventional tunneling that follows integral-order Bessel function ${{B}_{0}\left(\frac{2{{\uppi}} Ad{k}_{0}}{{\varLambda }_{\text{FL}}}\right)}^{2}$ for dynamic localization^38–40^ and ${{B}_{N}\left(\frac{2{{\uppi}} Ad{k}_{0}}{{\varLambda }_{\text{FL}}}\right)}^{2}$ for spreading^35–37^.

## Discussion

In summary, we report the first visual observation of BOs in photonic Floquet lattices and the investigation of photonic FBOs. In addition to the above-discussed cases with a harmonic modulation, we emphasize that FBOs occur for arbitrary Floquet engineering *M*(*z*) far beyond harmonic modulation. We experimentally verified photonic FBOs in Supplementary Note 4 with the three types of ${\partial }_{z}M(z)$, i.e. smooth function, nonsmooth continuous function, and discontinuous function.

Photonic FBOs are essentially a coherent phenomenon that can readily contribute to diverse platforms. As special cases of FBOs, conventional SBOs have been extended to ultracold atoms^24,25^, synthetic frequency lattices^15,29^, and quantum walks^31^. The exotic properties of FBOs can also be extended to these rapidly developing fields and may offer new insight into wide potential applications in high-efficiency frequency conversion, precision measurement, and wave manipulation^54^. For instance, the fractal spectrum of FBOs suggests that the FBO period is ultrasensitive to the ratio *Λ*_BO_/*Λ*_FL_, which may provide a new protocol for sensing.

Furthermore, the proposed FBOs may also contribute to fundamental research. Recently, space-time crystals have attracted interest because of their exotic oblique Brillouin zone^33,34^. In space-time crystals, the so-called FBOs (referring particularly to oscillations in time and space) arise from the periodic repetitions of Floquet dispersion. As a specific analogy to FBOs in space-time crystals, our proposed exact FBOs arise from the collapse of Floquet dispersion, and the corresponding visual observation is a cornerstone for the further development of space-time crystals.

## Materials and methods

### Sample fabrication

Our samples were fabricated inside a 90-mm-long polished fused silica substrate (Corning 7980) by a customized femtosecond-laser-writing system (Newport Corporation). See details in Supplementary Note 2.

### Fluorescence imaging characterization

A linearly polarized beam (TEM_00_ > 95%) at 633 nm wavelength from a 15 mW He-Ne laser (HNL150LB, Thorlabs) was employed for the single-site excitation and broad-beam excitation. Waveguide fluorescence microscopy was employed to directly visualize the light evolution in our samples. See details in Supplementary Note 3.

### Supplementary information

Supplementary File

## Data Availability

The experimental data supporting our findings of this study are available within the article and the Supplementary Information. All raw data are available from the corresponding authors upon reasonable request.
