# Supplementary material for: Visual observation of photonic Floquet–Bloch oscillations
Source: Light Sci Appl. 2024 Apr 28;13:99. doi: 10.1038/s41377-024-01419-z (PMC11056368; doi:10.1038/s41377-024-01419-z)
Supplement: Supplementary file 1 — Supplementary File [file 41377_2024_1419_MOESM1_ESM.pdf]

Supplementary Information for “Visual observation of photonic Floquet–Bloch oscillations”

Zhen Zhang,[1,3] Yuan Li,[2,3] Xiankai Sun,[2,*] and Xuewen Shu[1,*]

[1]*Wuhan National Laboratory for Optoelectronics and School of Optical and Electronic Information, Huazhong University of Science and Technology, Wuhan, Hubei 430074, China*

[2]*Department of Electronic Engineering, The Chinese University of Hong Kong, Shatin, New Territories, Hong Kong SAR, China*

[3]*These authors contributed equally to this work*

[*]*Corresponding author: xksun@cuhk.edu.hk (X. Sun); xshu@hust.edu.cn (X. Shu)*

Contents

Supplementary Note 1: Theory of photonic Floquet–Bloch oscillations 2

Theorem 1 & Proof... 2

Theorem 2 & Proof... 4

Supplementary Note 2: Sample fabrication 6

Supplementary Note 3: Fluorescence imaging characterization 7

Waveguide excitation 7

Waveguide fluorescence microscopy 7

Characterization process.. 8

Supplementary Note 4: Floquet–Bloch oscillations beyond harmonic modulation............. 11

Supplementary Note 5: Fractal spectrum.. 13

Supplementary Note 6: Observation of fractional Floquet tunneling 15

References... 19

Supplementary Note 1: Theory of photonic Floquet–Bloch oscillations

In this section, we present the detailed derivation of photonic Floquet–Bloch oscillations. To derive the corresponding results, we start the analysis by following the coupled equations derived in the manuscript:

$$i\frac{\partial a_m}{\partial z} = -c_0(a_{m-1} + a_{m+1}) - \frac{k_0 m F(z) d}{n_0} a_m \tag{S1}$$

where $a_m = a_m(z)$ is the amplitude of guided mode $|m\rangle$ in the mth waveguide and c_0 is the coupling constant between the nearest-neighbor waveguides.

In solving Eq. (S1), one performs a discrete Fourier transform over the site label m as

$$a_{k_x}(z) = \sum_m e^{-ik_x dm} a_m(z) \tag{S2}$$

and the form of Eq. (S1) in reciprocal space is expressed as

$$\frac{\partial a_{k_x}}{\partial z} = 2ic_0\cos(k_x)a_{k_x} + \frac{k_0 F(z) d}{n_0}\frac{\partial a_{k_x}}{\partial k_x} \tag{S3}$$

Following the method considered by Houston in the context of crystal electrons exposed to an electric field, we reconstructed the wave functions[1]:

$$\left|\psi_{m,k_x}(z)\right\rangle = \exp\left\{-\frac{in_0}{k_0}\int_0^z \beta_z[k_x(\tau)]d\tau\right\}\left|\psi_{m,k_x(z)}\right\rangle\Bigg\} \tag{S4}$$

where $\left|\psi_{m,k_x(z)}\right\rangle = \frac{1}{\sqrt{N}}\sum_m e^{ik_x(z)dm}|m\rangle$ and the transformation $k_x(z) = k_x(0) + \frac{k_0}{n_0}\int_0^z F(\tau)d\tau$. What Houston considered is a method of characteristics in mathematics, allowing one reduces a partial differential equation (S1) to a first-order ordinary differential equation:

$$\frac{da[z, k_x(z)]}{dz} = \left[2ic_0\cos(k_x)a_{k_x} + \frac{k_0 F(z) d}{n_0}\right]a[z, k_x(z)] \tag{S5}$$

with the explicit solution $a[z, k_x(z)] = \exp\{-i\int_0^z \beta_z[k_x(\tau)]d\tau\}a[0, k_x(0)]$ and single-band band structure $2c_0\cos(k_x d)$ serves as $\beta_z(k_x)$.

In order to carry out the two essential conclusions we draw in the main manuscript, we propose two theorems and corresponding proof as below:

Theorem 1

Floquet lattice with period Λ_{FL} in a linear tilted potential with corresponding BOs period Λ_{BO} can be mapped onto another Floquet lattice with a period Λ_{FBO} of the extended least common multiple of Λ_{FL} and Λ_{BO}.

Proof

When the ratio $\Lambda_{BO}/\Lambda_{FL}$ is a rational number, i.e., $\Lambda_{BO}/\Lambda_{FL}$ can be expressed as $\Lambda_{BO}/\Lambda_{FL} = Q/P$ where Q and P are mutually prime integers, we can define an extended least common multiple of Λ_{BO} and Λ_{FL} as the smallest rational number which is a multiple of both. The extended least common multiple of Λ_{BO} and Λ_{FL} satisfies the following equation: $\mathrm{LCM}(\Lambda_{BO}, \Lambda_{FL}) = P\Lambda_{BO} = Q\Lambda_{FL}$.

For the first step, we prove that single-band dispersion $\beta_z[k_x(z)]$ is a z-periodic function with a

period $\Lambda_{\rm FBO}$ = LCM($\Lambda_{\rm BO}$, $\Lambda_{\rm FL}$). Note that the function $\beta_z[k_x(z)] = \cos\left[k_x(0)d + \frac{2\pi z}{\Lambda_{\rm BO}} - k_0 d\partial_\tau M(\tau)|_0^z\right]$, where $\partial_z M(z)$ is a periodic function with period $\Lambda_{\rm FL}$. Obviously, for all values of z in the domain, the function $\beta_z[k_x(z)]$ satisfies the following equations:

$$\begin{aligned}\beta_z[k_x(z+\Lambda_{\rm FBO})] &= \cos\left[k_x(0) + \frac{2\pi(z+P\Lambda_{\rm BO})}{\Lambda_{\rm BO}} - k_0 d\partial_\tau M(\tau)|_0^{z+Q\Lambda_{\rm FL}}\right] \\ &= \cos\left[k_x(0) + \frac{2\pi z}{\Lambda_{\rm BO}} + 2\pi P - k_0 d\partial_\tau M(\tau)|_0^z\right] \\ &= \beta_z[k_x(z)]\end{aligned} \tag{S6}$$

then $\beta_z[k_x(z)]$ is a z-periodic function with a period $\Lambda_{\rm FBO}$ = LCM($\Lambda_{\rm FL}$, $\Lambda_{\rm BO}$) according to the definition of a periodic function.

For the second step, we prove that the integral of $\beta_z[k_x(z)]$ can be expressed as the sum of a linear function and a periodic function, i.e., $\int_0^z \beta_z[k_x(\tau)]d\tau = \varepsilon(k_x)z + P(z)$, where $P(z) = P(z + \Lambda_{\rm FBO})$ is a z-periodic function with a period $\Lambda_{\rm FBO}$.

We suppose that $B(z)$ is the integral of $\beta_z[k_x(z)]$, i.e., $B(z) = \int_0^z \beta_z[k_x(\tau)]d\tau$. We consider a function $P(z)$ by letting $P(z) = B(z) - \varepsilon(k_x)z$, where $\varepsilon(k_x) = \frac{1}{\Lambda_{\rm FBO}}\int_0^{\Lambda_{\rm FBO}} \beta_z[k_x(\tau)]d\tau$ is a z-independent term.

Since $\beta_z[k_x(z)]$ is a z-periodic function with a period $\Lambda_{\rm FBO}$, one has

$$\int_z^{z+\Lambda_{\rm FBO}} \beta_z[k_x(\tau)]d\tau = \int_0^{\Lambda_{\rm FBO}} \beta_z[k_x(\tau)]d\tau \tag{S7}$$

It follows that

$$\begin{aligned}P(z+\Lambda_{\rm FBO}) - P(z) &= B(z+\Lambda_{\rm FBO}) - B(z) + \varepsilon(k_x)z - \varepsilon(k_x)(z+\Lambda_{\rm FBO}) \\ &= \int_0^{z+\Lambda_{\rm FBO}} \beta_z[k_x(\tau)]d\tau - \int_0^z \beta_z[k_x(\tau)]d\tau - \varepsilon(k_x)\Lambda_{\rm FBO} \\ &= \int_z^{z+\Lambda_{\rm FBO}} \beta_z[k_x(\tau)]d\tau - \varepsilon(k_x)\Lambda_{\rm FBO} \\ &= \int_0^{\Lambda_{\rm FBO}} \beta_z[k_x(\tau)]d\tau - \int_0^{\Lambda_{\rm FBO}} \beta_z[k_x(\tau)]d\tau \\ &\equiv 0\end{aligned} \tag{S8}$$

That is, P(z) is a z-periodic function with a period $\Lambda_{\rm FBO}$ = LCM($\Lambda_{\rm FL}$, $\Lambda_{\rm BO}$). As a result, the integral of $\beta_z[k_x(z)]$ can be expressed as the sum of a linear function and a periodic function, i.e., $\int_0^z \beta_z[k_x(\tau)]d\tau = \varepsilon(k_x)z + P(z)$.

For the final step, as derived in manuscript, we reconstruct the Houston function as

$$\left|\psi_{m,k_x}(z)\right\rangle = \exp\left[-\frac{iz}{k_0}\varepsilon(k_x)\right]\left|u_{m,k_x(z)}\right\rangle \tag{S9}$$

where $\left|u_{m,k_x(z)}\right\rangle = \exp\left\{-\frac{i}{k_0}\int_0^z \beta_z[k_x(\tau)] - \varepsilon(k_x)d\tau\right\}\left|\psi_{m,k_x(z)}\right\rangle = \exp\left[-\frac{iP(z)}{k_0}\right]\left|\psi_{m,k_x(z)}\right\rangle$. Note that $\left|u_{m,k_x(z)}\right\rangle = \left|u_{m,k_x(z+\Lambda_{\rm FBO})}\right\rangle$ is a periodic function and thus Eq. (S9) has the same form as Floquet states. As a result, the Floquet lattice with modulation period $\Lambda_{\rm FL}$ in a linear tilted potential with corresponding BOs period $\Lambda_{\rm BO}$ can be also mapped onto another Floquet lattice with a period $\Lambda_{\rm FBO}$ of the extended least common multiple (LCM) of $\Lambda_{\rm FL}$ and $\Lambda_{\rm BO}$, where $\varepsilon(k_x)$ serves as Floquet dispersion.

Theorem 2

The existence of Floquet–Bloch oscillations is protected by the periodicity of Floquet engineering (or time translation symmetry) when $\Lambda_{FL} \neq N\Lambda_{BO}$ (N is any positive integer), while spreading in general occurs when $\Lambda_{FL} = N\Lambda_{BO}$.

Proof

As discussed in Theorem 1, the Floquet dispersion $\varepsilon(k_x) \equiv \frac{1}{\Lambda_{\text{FBO}}}\int_0^{\Lambda_{\text{FBO}}} \beta_z[k_x(\tau)]d\tau$ provides the effective transport properties over one period Λ_{FBO}. To illustrate the Bloch oscillations in the Floquet lattice, we begin with the analysis of Floquet dispersion:

$$\begin{aligned}\varepsilon(k_x) &\equiv \frac{1}{\Lambda_{\text{FBO}}}\int_0^{\Lambda_{\text{FBO}}} \beta_z[k_x(\tau)]d\tau \\ &= \frac{1}{\Lambda_{\text{FBO}}}\int_0^{\Lambda_{\text{FBO}}} \cos\left[k_x(0)d + \frac{2\pi\tau}{\Lambda_{\text{BO}}} - k_0 d\partial_z M(z)|_0^{\tau}\right] d\tau\end{aligned} \tag{S10}$$

Considering the integral term, one arrives at the expression:

$$\begin{aligned}&\int_0^{\Lambda_{\text{FBO}}} \cos\left[k_x(0)d + \frac{2\pi\tau}{\Lambda_{\text{BO}}} - k_0 d\partial_z M(z)|_0^{\tau}\right] d\tau \\ &= \int_0^{\Lambda_{\text{FL}}} \cos\left[k_x(0)d + \frac{2\pi\tau}{\Lambda_{\text{BO}}} - k_0 d\partial_z M(z)|_0^{\tau}\right] d\tau + \ldots + \int_{(Q-1)\Lambda_{\text{FL}}}^{Q\Lambda_{\text{FL}}} \cos\left[k_x(0)d + \frac{2\pi\tau}{\Lambda_{\text{BO}}} - k_0 d\partial_z M(z)|_0^{\tau}\right] d\tau\end{aligned} \tag{S11}$$

We replace τ with $(n\Lambda_{\text{FL}} - \tau)$ for the nth terms and the above integral has the form of

$$\begin{aligned}&\int_0^{\Lambda_{\text{FBO}}} \cos\left[k_x(0)d + \frac{2\pi\tau}{\Lambda_{\text{BO}}} - k_0 d\partial_z M(z)|_0^{\tau}\right] d\tau \\ &= \int_0^{\Lambda_{\text{FL}}} \cos\left[2\pi\frac{P}{Q} + k_x(0)d - \frac{2\pi\tau}{\Lambda_{\text{BO}}} - k_0 d\partial_z M(z)|_0^{-\tau}\right] d\tau + \ldots + \int_0^{\Lambda_{\text{FL}}} \cos\left[2\pi n\frac{P}{Q} + k_x(0)d - \frac{2\pi\tau}{\Lambda_{\text{BO}}} - k_0 d\partial_z M(z)|_0^{-\tau}\right] d\tau \\ &= \sum_{n=1}^{Q}\cos\left(\frac{2\pi P}{Q}n\right)\int_0^{\Lambda_{\text{FL}}} \cos\left[k_x(0)d - \frac{2\pi\tau}{\Lambda_{\text{BO}}} - k_0 d\partial_z M(z)|_0^{-\tau}\right] d\tau - \sum_{n=1}^{Q}\sin\left(\frac{2\pi P}{Q}n\right)\int_0^{\Lambda_{\text{FL}}} \sin\left[k_x(0)d - \frac{2\pi\tau}{\Lambda_{\text{BO}}} - k_0 d\partial_z M(z)|_0^{-\tau}\right] d\tau\end{aligned} \tag{S12}$$

Note that the value of sum $\sum_{n=1}^{Q}\cos\left(\frac{2\pi P}{Q}n\right)$ and $\sum_{n=1}^{Q}\sin\left(\frac{2\pi P}{Q}n\right)$ depends on the ratio P/Q, which should be analyzed from two aspects:

$$\begin{cases} \text{i.} \quad \begin{aligned}\textstyle\sum_{n=1}^{Q}\cos\left(\frac{2\pi P}{Q}n\right) &= \frac{\sin(P\pi)}{\sin(P\pi/Q)}\cos\left(\frac{P\pi}{Q}+P\pi\right) \equiv 0 \\ \textstyle\sum_{n=1}^{Q}\sin\left(\frac{2\pi P}{Q}n\right) &= \frac{\sin(P\pi)}{\sin(P\pi/Q)}\sin\left(\frac{P\pi}{Q}+P\pi\right) \equiv 0\end{aligned} & \text{when } P/Q \neq \text{any integers } N, \\ \text{ii.} \quad \begin{aligned}\textstyle\sum_{n=1}^{Q}\cos\left(\frac{2\pi P}{Q}n\right) &\equiv Q \equiv \frac{\Lambda_{\text{FBO}}}{\Lambda_{\text{FL}}} \\ \textstyle\sum_{n=1}^{Q}\sin\left(\frac{2\pi P}{Q}n\right) &\equiv 0\end{aligned} & \text{when } P/Q = \text{any integers } N. \end{cases} \tag{S13}$$

For case i, i.e., $\Lambda_{\text{FL}} \neq N\Lambda_{\text{BO}}$, there is a complete cancellation of all orders of diffraction, resulting in a flat Floquet band structure $\varepsilon(k_x) \equiv 0$. Under this circumstance, the state experiences a periodic motion and relocalizes to the initial state after propagating a period Λ_{FBO}, i.e., $\left|\psi_{m,k_x}(z + \Lambda_{\text{FBO}})\right\rangle = \left|\psi_{m,k_x}(z)\right\rangle$. We call this phenomenon Floquet–Bloch oscillations since this new type of Bloch oscillations is a combined phenomenon of Floquet engineering and Bloch oscillation. Note that the complete cancellation is guaranteed by the periodicity $M(z) = M(z + \Lambda_{\text{FL}})$ and no specific modulation $M(z)$ is required.

For case ii, i.e., $\Lambda_{\text{FL}} = N\Lambda_{\text{BO}}$, the Floquet band structure $\varepsilon(k_x) \equiv \frac{2c_0}{\Lambda_{\text{FL}}}\int_0^{\Lambda_{\text{FL}}} \cos\Big[k_x(0)d - \frac{2\pi\tau}{\Lambda_{\text{BO}}} -$

$k_0 d\partial_z M(z)|_0^{-\tau}\Big] d\tau$ in general is no longer flat. Under that circumstance, the state experiences spreading with a rescaled dispersion $\varepsilon(k_x)$. Note that spreading exhibits its similarity to the well-known dynamic localization since $\int_0^{\Lambda_{\mathrm{FL}}} \cos\left[k_x(0)d - \frac{2\pi\tau}{\Lambda_{\mathrm{BO}}} - k_0 d\partial_z M(z)|_0^{-\tau}\right] d\tau$ can be zero with a specific $M(z)$.

Supplementary Note 2: Sample fabrication

Our samples were fabricated inside a 90-mm-long polished fused silica substrate (Corning 7980) by a customized femtosecond-laser-writing system (Newport Corporation). The second harmonic generation by a Yb-based amplified femtosecond laser source (Spirit 1040-8-SHG) provides circularly polarized laser pulses with 220-fs pulse duration at the central wavelength of 520 nm. The laser beam was reshaped using a 5.5-mm-diameter circular aperture and subsequently focused at 150 μm below the substrate surface using a 20× objective (RMS20X, Olympus). Here the aperture was employed to filter out irregular edges of the laser beam for suppressing the irregularities in the shape and energy distribution of the focused spot, and to expand the focal spot accordingly for averting laser-induced damage, thereby enhancing the performance of waveguides, including mode field profile optimization and propagation loss reduction. At the same time, a 3-axis ultra-precision motorized stage (translation stages, XMS100 and NPXYZ100SG-D; motion controller, XPS-Q) was employed to translate the substrate with respect to the laser beam and the laser-exposed zone following the designed trajectory formed the guiding core of waveguides. The ultra-precision stage with bi-directional repeatability (±40nm) provides robust motion with accuracy on the most complex trajectories and the deviations between the designed trajectory and the fabrication trajectory is negligible. With the optimized irradiation parameters (360 nJ pulse energy, 20 kHz repetition rate, and 10 mm/s translation speed), the straight waveguide supports a well-confined fundamental mode at the wavelength of 633 nm and the corresponding intrinsic loss is estimated to be 0.33 dB/cm.

Uniformity and consistency of the fabricated samples are the keys for achieving excellent agreement between experiments and simulations, where accuracy, uniformity, and reproducibility of the fabrication play important roles. We have made elaborate efforts to calibrate the fabrication process including:

1. **Exposure time correction:** The bending trajectory of each waveguide is evenly broken down into 9000 discrete straight lines in the z direction. The translation speed remains constant along each straight line, resulting in a homogeneous waveguide.
2. **Horizontal adjustment**: With the aid of an imaging system, the laser focal plane was monitored. The femtosecond laser is then aligned perpendicular to the substrate surface to fabricate waveguide arrays of consistent depth.
3. **Power compensation**: A power meter (Model 1918-R, Newport) was used for monitoring the average power of the laser pulse and power compensation was presented, ensuring the waveguide array is reproducible.

Supplementary Note 3: Fluorescence imaging characterization

The experimental setup for sample characterization is depicted in Fig. S1. The sample characterization can be divided into two parts: waveguide excitation and waveguide fluorescence microscopy.

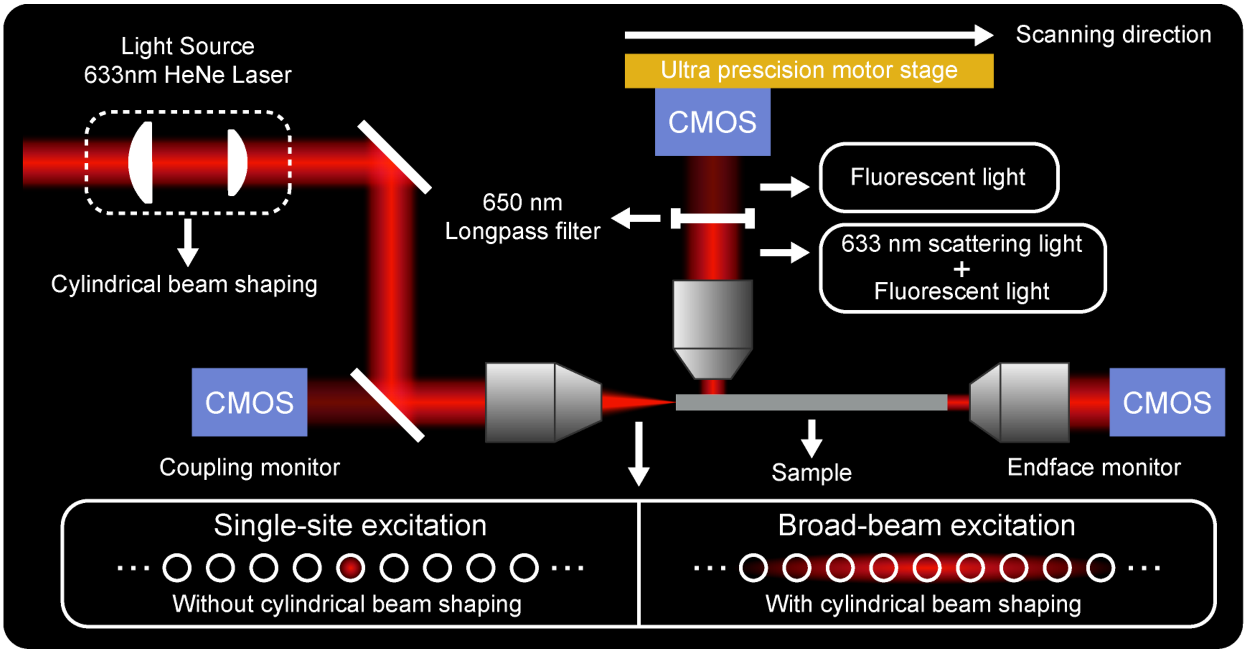

Fig. S1. Experimental setup for sample characterization.

Waveguide excitation

A linearly polarized beam (TEM_{00}>95%) at 633 nm wavelength from a 15 mW He-Ne laser (HNL150LB, Thorlabs) was employed for the single-site excitation and broad-beam excitation. Note that the polarization direction of laser beam is aligned with the major axis of the elliptical waveguide, ensuring the preservation of polarization mode. Indeed, the polarization of the excitation does not affect the photonic Floquet–Bloch oscillations, where the vectorial features of the optical waves do not appear. The sample was positioned on a 3-axis, tilt, and rotary stage, which was used to execute precise alignment of lateral (x, y, z) and angular (pitch, yaw and roll) degree-of-freedoms for improving coupling coefficient. The input (output) facet of the waveguide array was imaged by a coupling (endface) monitor for aiding coupling alignment.

In the case of single-site excitation, we launched a circular Gaussian beam into the central waveguide of the arrays through a 20 × objective (0.4 NA). The diameter of the focused beam at the input facet was approximately 4 μm that was much smaller than the waveguide spacing, avoiding unwanted excitation of several waveguides.

In the case of broad-beam excitation, we reshaped the x-component of the laser beam with a cylindrical lens pair and then launched the beam into our sample through a 5 × objective (0.1 NA). At the input facet of the waveguide array, approximately 7 waveguides were excited by an elongated Gaussian beam (~100 μm width and ~15 μm height, at its center).

Waveguide fluorescence microscopy

We used waveguide fluorescence microscopy to directly visualize the light evolution in our

samples. Femtosecond laser exposure in fused silica with a high content of OH generates homogeneous spatial distributed nonbridging oxygen hole centers (NBOHCs). With the excitation of the He-Ne laser (633 nm), these NBOHCs emit fluorescence at around 650 nm. Since the fluorescence intensity is proportional to the intensity of the light propagating within the arrays, the top-view fluorescent signal provides a reliable method to quantify the light evolution.
Scattering light arising from insufficiently coupling and leaking light arising from bending structure may largely influence the fluorescence imaging quality. In our experiments, the scattering and leaking light are barely observed in fluorescence imaging for the following two aspects:

1. The fluorescent color centers generated by femtosecond laser writing were exclusively located inside the laser-modified zone, i.e., the guiding core of waveguides. With an employed long-pass filter, the scattering of excitation light is almost blocked, and only fluorescent light emitted from the waveguides can be observed.
2. In our experiments, the guiding structure is gradual (curvature~10^{-6}) so that the guided light barely couples into the unbound radiative modes. In this connection, the leaking light is negligible compared to the fluorescent signals from light evolution.

Characterization process

In this section, we outline the characterization process that is used to extract the light evolution. The detailed characterization process is presented in Fig. S2, which consists of three parts:

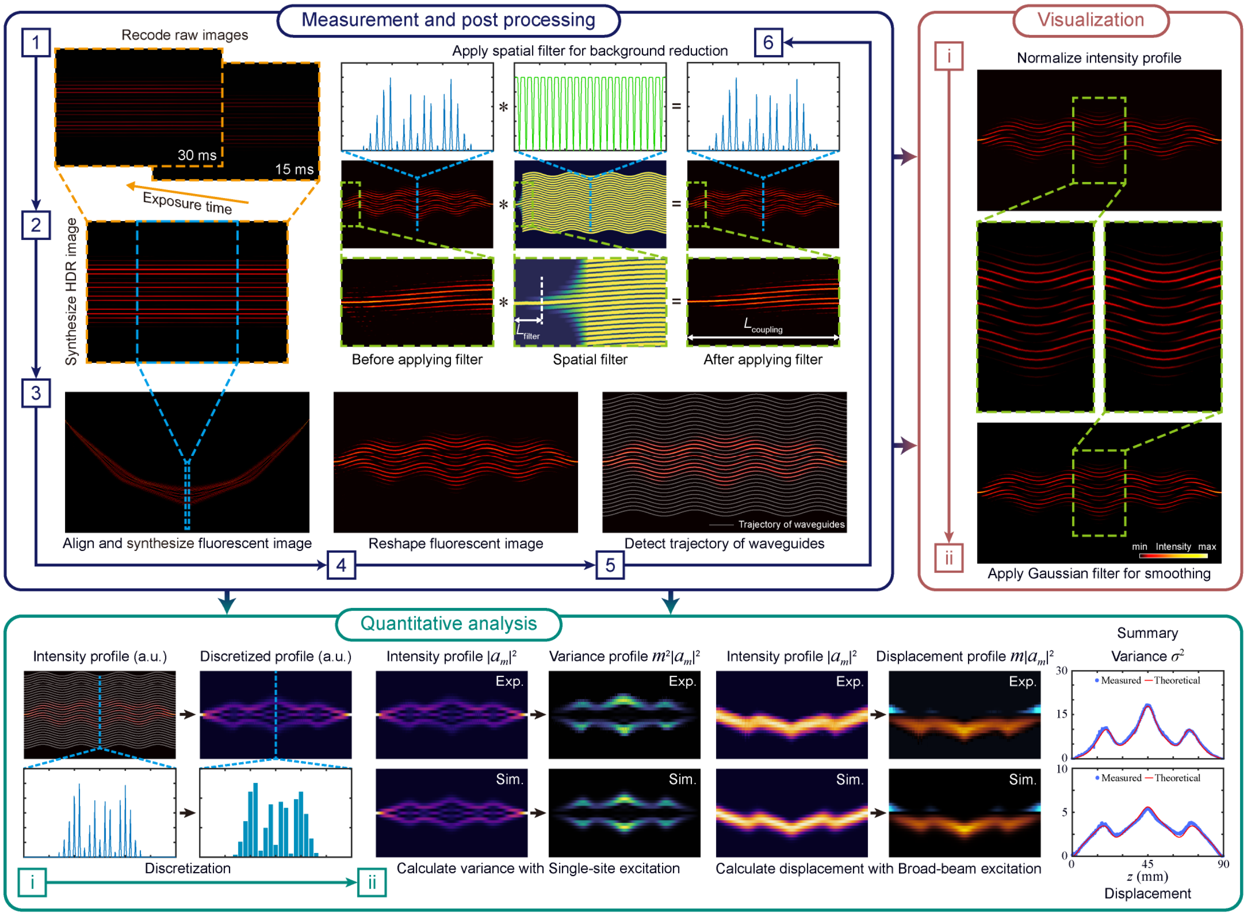

Fig. S2. Detailed characterization process.

measurement and post processing, visualization, and quantitative analysis. The top-view fluorescent signal is recorded and post-processed, then visualized and quantitative analyzed individually.

For the measurement and post processing, we apply the following steps:

1 **Record raw data:**

The top-view fluorescent signal emitted from the sample was collected through a 10× objective (0.25 NA) and recorded by a CMOS sensor (IMX178, Sony). By translating the top-view imaging system with an ultra-precision motor stage by a uniform interval (approximately half of the image length), we scanned over the sample along the *z* direction and recorded multiple fluorescent images at different exposure time.

2 **Synthesize HDR image:**

The red channel of the recorded fluorescent images was extracted and converted into grayscale, which collects fluorescent intensity information. After corrected for the exposure time and camera response function, multiple fluorescent images of the same scene were synthetized into a high dynamic range (HDR) image (recolored). In this way, the synthetized HDR image has an excellent dynamic range, that prevents weak signals from being concealed.

3 **Align and synthesize fluorescent image:**

To prevent from affecting by image distortion around the edges of images, each HDR image are cropped into half (dashed blue box). Subsequently, the cropped HDR images were compressed in the *z* direction and aligned according to feature around the edges and then stitched in sequence to yield a fluorescent image of the entire light evolution in the sample.

4 **Reshape fluorescent image:**

To visualize the light evolution more intuitively, a coordinate transformation that maps circular arcs into straight lines was applied to reshape the fluorescence image, where each slice along the *z* direction was digitally shifted.

5 **Detect trajectory of waveguides:**

We tracked the trajectory of the central waveguide in the transformed coordinates based on the features of the light intensity distribution at each slice. Subsequently, we created an array of detected trajectories with identical spacing.

6 **Apply spatial filter for background reduction:**

Due to the imperfect coupling, a slight amount of uncoupled light emits fluorescence around the input facet. To mitigate this impact, a digital spatial filter was utilized to filter out those of the uncoupled light (dashed green box) and keep only the signals of light evolution. Note that the filter length L_{filter} is much shorter than coupling length L_{coupling} so that the light evolution can be considered unaffected (dashed blue box). The splice of the filter is a set of Tukey (tapered cosine) windows (green lines) with a width of approximately 15.8 μm, and the number of windows was determined for background reduction. The fluorescent imaging after applying the filter was given by the Hadamard product of the fluorescent imaging before applying the filter and the spatial filter matrix, where the sign * stands for the Hadamard multiplication.

For the visualization, we applied the following two steps:

ⓘ **Normalize intensity profile:**

The intensity of light evolution was normalized to unity along the propagation direction so that the fluorescence intensity decay and propagation losses of the waveguides were compensated.

ⓘⓘ **Apply Gaussian filter for smoothing:**

To visualize the light evolution more intuitively, we applied a Gaussian filter to smooth and slightly broaden the fluorescence that exclusively located inside the guiding core of waveguides. As shown in the dashed green box, the fluorescence intensity profile after filtering is much easier to be recognized compared to that before filtering.

For the quantitative analysis, we apply the following two steps:

ⓘ **Discretization:**

Since the fluorescence intensity is proportional to the intensity of the light propagating within the arrays, the fluorescence intensity profile provides a reproduction of the light evolution. According to the detected trajectory, we summed up the fluorescence intensity around each waveguide as an experimental measurement of light evolution under tight-binding approximation. Here we display the normalized discretized intensity profile ($\sum_m |a_m|^2 = 1$).

ⓘⓘ **Calculate variance/displacement profile:**

With the discretized intensity profile of light, one may calculate the measured variance/displacement profile. As shown in the figure, we take FBOs ($\Lambda_{\mathrm{BO}}/\Lambda_{\mathrm{FL}} = 4/3$) under single-site and broad-beam excitations as two examples. According to the definition, the variance $\sigma^2(z) = \frac{\sum_m m^2|a_m|^2}{\sum_m |a_m|^2}$ [displacement $x(z) = \frac{\sum_m md|a_m|^2}{\sum_m |a_m|^2}$] is given by summing up each row of variance/displacement profile. For both single-site and broad-beam excitations, the discretized intensity profile and quantitative analyses have excellent agreement with the respective simulation results.

Supplementary Note 4: Floquet–Bloch oscillations beyond harmonic modulation

In the main manuscript, we employ harmonic modulation [$M(z) = \cos(2\pi z/\Lambda_{\mathrm{FL}})$] to illustrate the similarity and difference between Floquet–Bloch oscillations and previous Bloch oscillations. As discussed above, Floquet–Bloch oscillations occur for arbitrary Floquet engineering $M(z)$, which is far beyond harmonic modulation. According to Eq. (5) in the main manuscript, the theoretical analysis shows that the Floquet dispersion $\varepsilon(k_x)$ of the Floquet function is affected by $\partial_z M(z)$. Therefore, Floquet engineering $M(z)$ in arbitrary form can be considered into three cases: (i) $\partial_z M(z)$ is a smooth function; (ii) $\partial_z M(z)$ is a nonsmooth continuous function; (iii) $\partial_z M(z)$ is a discontinuous function. In this section, we experimentally verified FBOs in the photonic lattices with the three types of $\partial_z M(z)$ as shown in Fig. S3. The photonic lattices follow the combined bending trajectories with circular bend radius R = 110.8 cm (corresponding to $\Lambda_{\mathrm{BO}} \sim 30$ mm), the modulation periods Λ_{FL} = 22.5 mm (corresponding to the ratio $\Lambda_{\mathrm{FL}}/\Lambda_{\mathrm{BO}} = 3/4$) and the modulation amplitude A = 13.5 μm.

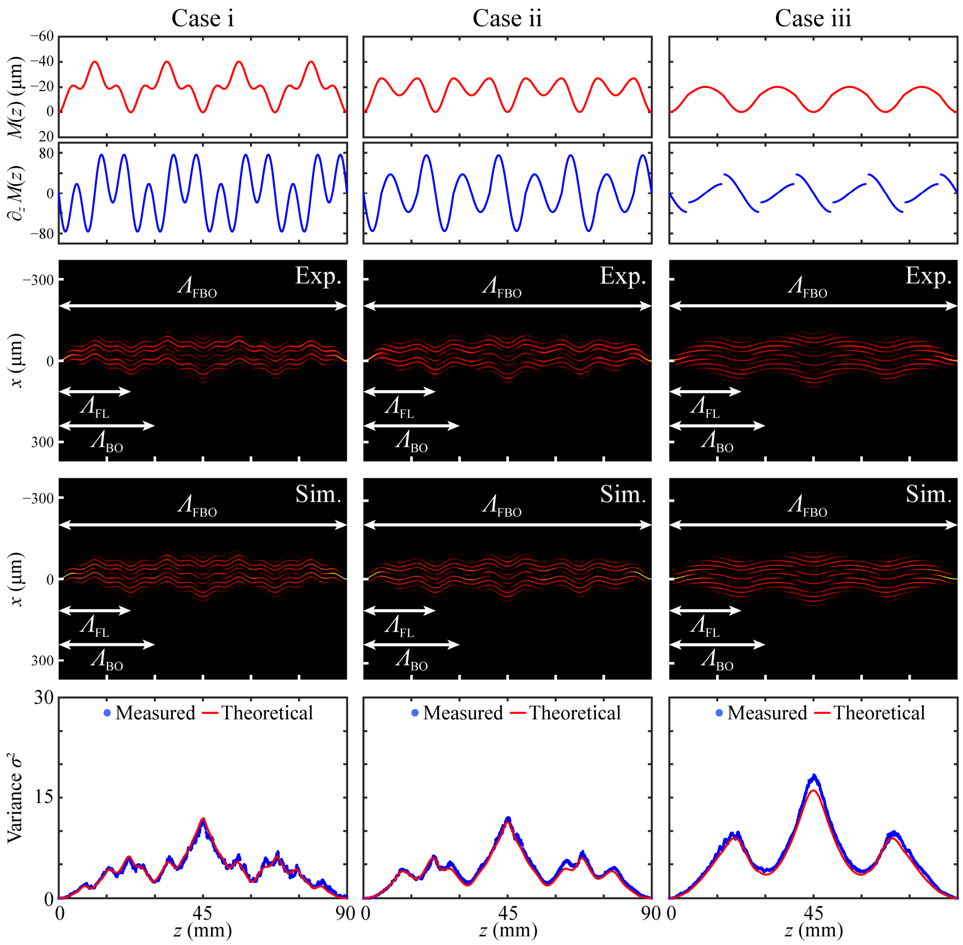

Fig. S3. Experimental visualization, simulation, and variance of the breathing modes in the photonic lattices under the three cases of Floquet engineering $M(z)$. (Case i) $\partial_z M(z)$ is a smooth function; (Case ii) $\partial_z M(z)$ is a nonsmooth continuous function; (Case iii) $\partial_z M(z)$ is a discontinuous function. In all the cases, the Floquet–Bloch oscillations occur with an identical FBO period $\Lambda_{\mathrm{FBO}} = \mathrm{LCM}(\Lambda_{\mathrm{FL}}, \Lambda_{\mathrm{BO}})$ = 90 mm.

For the smooth function $\partial_z M(z)$ (case i), the Floquet engineering $M(z)$ employed in the photonic lattices is expressed as

$$M(z) = A\cos(2\pi z/\Lambda_{\mathrm{FL}}) + \frac{A}{2}\cos(6\pi z/\Lambda_{\mathrm{FL}}) \tag{S14}$$

For the nonsmooth continuous function $\partial_z M(z)$ (case ii), the Floquet engineering $M(z)$ employed in the photonic lattices is expressed as

$$M(z) = \begin{cases} A\cos\left(\dfrac{4\pi z}{\Lambda_{\mathrm{FL}}}\right), & N\Lambda_{\mathrm{FL}} \le z < N\Lambda_{\mathrm{FL}} + \dfrac{\Lambda_{\mathrm{FL}}}{4} \\ \dfrac{A}{2}\left[\cos\left(\dfrac{4\pi z}{\Lambda_{\mathrm{FL}}}\right) - 1\right], & N\Lambda_{\mathrm{FL}} + \dfrac{\Lambda_{\mathrm{FL}}}{4} \le z < N\Lambda_{\mathrm{FL}} + \dfrac{3\Lambda_{\mathrm{FL}}}{4} \\ A\cos\left(\dfrac{4\pi z}{\Lambda_{\mathrm{FL}}}\right), & N\Lambda_{\mathrm{FL}} + \dfrac{3\Lambda_{\mathrm{FL}}}{4} \le z < N\Lambda_{\mathrm{FL}} + \Lambda_{\mathrm{FL}} \end{cases} \tag{S15}$$

For the discontinuous function type $\partial_z M(z)$ (case iii), the Floquet engineering $M(z)$ employed in the photonic lattices is expressed as

$$M(z) = \begin{cases} A\cos\left(\dfrac{2\pi z}{\Lambda_{\mathrm{FL}}}\right), & N\Lambda_{\mathrm{FL}} \le z < N\Lambda_{\mathrm{FL}} + \dfrac{\Lambda_{\mathrm{FL}}}{4} \\ \dfrac{A}{2}\cos\left(\dfrac{2\pi z}{\Lambda_{\mathrm{FL}}}\right), & N\Lambda_{\mathrm{FL}} + \dfrac{\Lambda_{\mathrm{FL}}}{4} \le z < N\Lambda_{\mathrm{FL}} + \dfrac{3\Lambda_{\mathrm{FL}}}{4} \\ A\cos\left(\dfrac{2\pi z}{\Lambda_{\mathrm{FL}}}\right), & N\Lambda_{\mathrm{FL}} + \dfrac{3\Lambda_{\mathrm{FL}}}{4} \le z < N\Lambda_{\mathrm{FL}} + \Lambda_{\mathrm{FL}} \end{cases} \tag{S16}$$

Under the single-site excitations, the experimental observations of evolution patterns, respective simulations, and extracted variances $\sigma^2(z)$ for the scenarios are depicted in Fig. S3. It is obvious that Floquet–Bloch oscillations occur for all three types of $\partial_z M(z)$ with an identical FBO period Λ_{FBO} = 90 mm, namely that FBOs occur for arbitrary Floquet engineering $M(z)$.

Supplementary Note 5: Fractal spectrum

In this section, we study the dependence of the ratio $\Lambda_{\mathrm{BO}}/\Lambda_{\mathrm{FBO}}$ on the ratio $\Lambda_{\mathrm{BO}}/\Lambda_{\mathrm{FL}}$. When the ratio $\Lambda_{\mathrm{BO}}/\Lambda_{\mathrm{FL}}$ is a rational number and $\Lambda_{\mathrm{FL}} \neq N\Lambda_{\mathrm{BO}}$, the theoretically predicted FBO period follows $\Lambda_{\mathrm{FBO}} = \mathrm{LCM}(\Lambda_{\mathrm{BO}}, \Lambda_{\mathrm{FL}})$ and otherwise the FBO period tends toward infinite. Therefore, the FBO period spectrum can be expressed as

$$\frac{\Lambda_{\mathrm{BO}}}{\Lambda_{\mathrm{FBO}}} = \begin{cases} \frac{1}{P}, & \text{if } \frac{\Lambda_{\mathrm{BO}}}{\Lambda_{\mathrm{FL}}} = \frac{Q}{P} \text{ with } Q \text{ and } P \text{ are mutually prime integers; } \Lambda_{\mathrm{FL}} \neq N\Lambda_{\mathrm{BO}}, \\ 0, & \text{if } \frac{\Lambda_{\mathrm{BO}}}{\Lambda_{\mathrm{FL}}} \text{ is irrational or } \Lambda_{\mathrm{FL}} = N\Lambda_{\mathrm{BO}}. \end{cases} \tag{S17}$$

One may find that the FBO period spectrum follows the Thomae's function, which is a fractal structure composed of infinite discrete peaks[2].

To experimentally verify our prediction, we employed the harmonic modulation $M(z) = A\cos(2\pi z/\Lambda_{\mathrm{FL}})$ as a modulation function. With this specific modulation and the single-site excitations, there is always a peak or a trough of extracted variances $\sigma^2(z)$ at $z = \Lambda_{\mathrm{FBO}}/2$. As a result, we can measure the value of $\Lambda_{\mathrm{FBO}}/2$ by characterizing the position of the peak or trough. Owing to the limited length of the sample, only the cases where $\Lambda_{\mathrm{BO}}/\Lambda_{\mathrm{FBO}}$ is greater than 1/6 can be verified experimentally. A set of photonic lattices with a fixed Bloch oscillation period Λ_{BO} = 30 mm and varied modulation periods Λ_{FL} were fabricated. The modulation periods Λ_{FL} are set to 25.71, 25, 24, 22.5, 21.43, 20, 18.75, 18, 17.14, 16.67, 16.36, and 15 mm, corresponding to the ratios $\Lambda_{\mathrm{BO}}/\Lambda_{\mathrm{FL}}$ = 7/6, 6/5, 5/4, 4/3, 7/5, 3/2, 8/5, 5/3, 7/4, 9/5, 11/6, and 2, respectively. Considering the radiation losses of waveguides, the modulation amplitude A that corresponds to the modulation periods Λ_{FL} in order are set to 15.43, 15, 14.4, 13.5, 12.86, 12, 11.25, 10.8, 10.29, 3.33, and 3 μm, respectively. The detailed results of evolution patterns, respective simulations, and extracted variances $\sigma^2(z)$ for the scenarios are depicted in Fig. S4. Note that the variances $\sigma^2(z)$ of experimental results are in excellent agreement with the simulation values, thereby confirming the precise values of the FBO periods Λ_{FBO}.

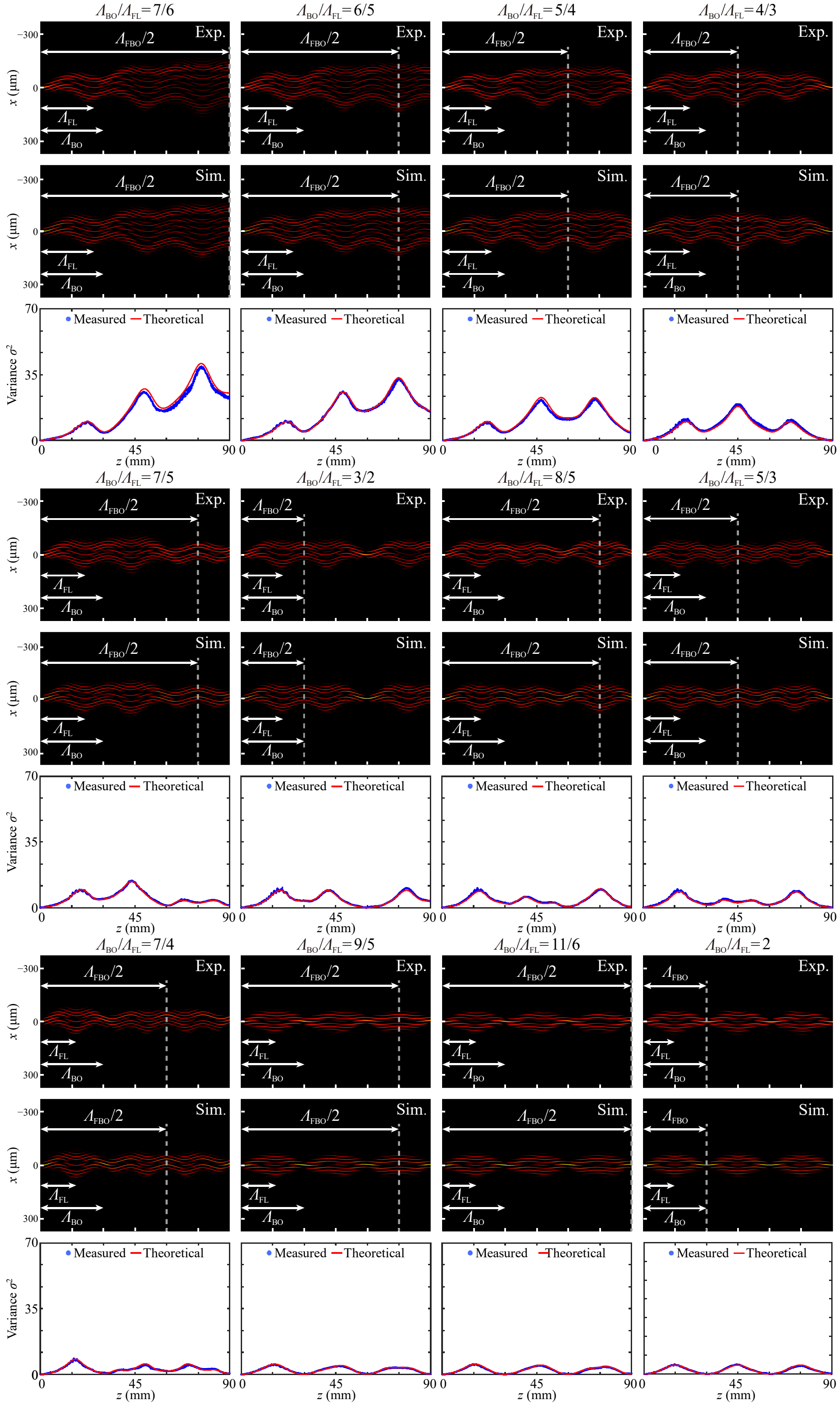

Fig. S4. Experimental visualization, simulation, and variance of the breathing modes for measuring the FBO periods. The ratio $\Lambda_{BO}/\Lambda_{FL}$ starts from 7/6 to 2, where experimental (Exp.) and simulated (Sim.) results are compared.

Supplementary Note 6: Observation of fractional Floquet tunneling

In the main manuscript, we defined the FBO amplitude as $\sigma^2(\Lambda_{\mathrm{FBO}}/2)$ and observe the fractional Floquet tunneling with the harmonic modulation $M(z) = A\cos(2\pi z/\Lambda_{\mathrm{FL}})$. In this section, we provide detailed theoretical analyses and additional experimental results.

To illustrate the fractional Floquet tunneling, we begin with a general form of the variance $\sigma^2(z)$ with the harmonic modulation:

$$\sigma^2(z) = 2c_0^2\left[G(z)^2 + H(z)^2\right] \tag{S18}$$

where $G(z) = \int_0^z \cos\left[\frac{2\pi\tau}{\Lambda_{\mathrm{BO}}} + \frac{2\pi A dk_0}{\Lambda_{\mathrm{FL}}}\sin\left(\frac{2\pi\tau}{\Lambda_{\mathrm{FL}}}\right)\right] d\tau$ and $H(z) = \int_0^z \sin\left[\frac{2\pi\tau}{\Lambda_{\mathrm{BO}}} + \frac{2\pi A dk_0}{\Lambda_{\mathrm{FL}}}\sin\left(\frac{2\pi\tau}{\Lambda_{\mathrm{FL}}}\right)\right] d\tau$. When $\Lambda_{\mathrm{BO}}/\Lambda_{\mathrm{FL}} = Q/P$ with Q and P are mutually prime integers, the FBO periods Λ_{FBO} is given by $\mathrm{LCM}(\Lambda_{\mathrm{BO}}, \Lambda_{\mathrm{FL}}) = P\Lambda_{\mathrm{BO}} = Q\Lambda_{\mathrm{FL}}$. Considering the change of variable $z = \Lambda_{\mathrm{FL}}\theta/(2\pi)$, one arrives at the expression of $G(\Lambda_{\mathrm{FBO}}/2)$ as

$$\begin{aligned} G\left(\frac{\Lambda_{\mathrm{FBO}}}{2}\right) &= \int_0^{\frac{\Lambda_{\mathrm{FBO}}}{2}} \cos\left[\frac{2\pi\tau}{\Lambda_{\mathrm{BO}}} + \frac{2\pi A dk_0}{\Lambda_{\mathrm{FL}}}\sin\left(\frac{2\pi\tau}{\Lambda_{\mathrm{FL}}}\right)\right] d\tau \\ &= \frac{\Lambda_{\mathrm{FL}}}{2\pi}\int_0^{Q\pi} \cos\left[\frac{P}{Q}\theta + \frac{2\pi A dk_0}{\Lambda_{\mathrm{FL}}}\sin(\theta)\right] d\theta \\ &= \frac{\Lambda_{\mathrm{FL}}}{2\pi}\int_0^{\pi} \cos\left[\frac{P}{Q}\theta + \frac{2\pi A dk_0}{\Lambda_{\mathrm{FL}}}\sin(\theta)\right] d\theta + \ldots + \frac{\Lambda_{\mathrm{FL}}}{2\pi}\int_{(Q-1)\pi}^{Q\pi} \cos\left[\frac{P}{Q}\theta + \frac{2\pi A dk_0}{\Lambda_{\mathrm{FL}}}\sin(\theta)\right] d\theta \end{aligned} \tag{S19}$$

We replace θ with $\theta - 2\pi(Q-1)/2$ for the odd Qth terms and with $\pi Q - \theta$ for the even Qth terms, and then the above integral has the form of

$$G\left(\frac{\Lambda_{\mathrm{FBO}}}{2}\right) = \sum_{n=1}^{Q} g_n \tag{S20}$$

and

$$g_n = \begin{cases} \frac{\Lambda_{\mathrm{FL}}}{2}\cos\left[\frac{P\pi(n-1)}{Q}\right] J_v\left(-\frac{2\pi A dk_0}{\Lambda_{\mathrm{FL}}}\right) - \frac{\Lambda_{\mathrm{FL}}}{2}\sin\left[\frac{P\pi(n-1)}{Q}\right] E_v\left(-\frac{2\pi A dk_0}{\Lambda_{\mathrm{FL}}}\right), & \text{if } n \text{ is odd,} \\ \frac{\Lambda_{\mathrm{FL}}}{2}\cos\left(\frac{P\pi n}{Q}\right) J_v\left(-\frac{2\pi A dk_0}{\Lambda_{\mathrm{FL}}}\right) + \frac{\Lambda_{\mathrm{FL}}}{2}\sin\left(\frac{P\pi n}{Q}\right) E_v\left(-\frac{2\pi A dk_0}{\Lambda_{\mathrm{FL}}}\right), & \text{if } n \text{ is even.} \end{cases} \tag{S21}$$

where

$$J_v(z) = \frac{1}{\pi}\int_0^{\pi} \cos\left[v\theta - z\sin(\theta)\right] d\theta \tag{S22}$$

and

$$E_v(z) = \frac{1}{\pi}\int_0^{\pi} \sin\left[v\theta - z\sin(\theta)\right] d\theta \tag{S23}$$

denote the classical Anger and Weber functions with an order $v = P/Q$, respectively[3]. The value of $G(\Lambda_{\mathrm{FBO}}/2)$ should be analyzed from two aspects:

$$G\left(\frac{\Lambda_{\mathrm{FBO}}}{2}\right) = \begin{cases} \text{i.} \quad \frac{\Lambda_{\mathrm{FL}}}{2}\frac{\sin\left[\frac{(Q-2)P\pi}{2Q}\right]\cos\left(\frac{P\pi}{2}\right)}{\sin\left(\frac{P\pi}{Q}\right)} J_v\left(-\frac{2\pi A dk_0}{\Lambda_{\mathrm{FL}}}\right) & \text{when } Q \text{ is even,} \\ \text{ii.} \quad \frac{\Lambda_{\mathrm{FL}}}{2}\frac{\sin(P\pi)}{\sin\left(\frac{P\pi}{Q}\right)} J_v\left(-\frac{2\pi A dk_0}{\Lambda_{\mathrm{FL}}}\right) & \text{when } Q \text{ is odd.} \end{cases} \tag{S24}$$

Since Q and P are mutually prime integers, P must be odd when Q is even, and thus $\cos(P\pi/2) = 0$. Therefore, it is proven that $G(\Lambda_{\mathrm{FBO}}/2) \equiv 0$, regardless of the value of P and Q. In a similar way,

one can find that the $H(\Lambda_{\mathrm{FBO}}/2)$ has the form of

$$H\left(\frac{\Lambda_{\mathrm{FBO}}}{2}\right) = \sum_{n=1}^{Q} h_n \tag{S25}$$

and

$$h_n = \begin{cases} \frac{\Lambda_{\mathrm{FL}}}{2}\cos\left[\frac{P\pi(n-1)}{Q}\right]E_v\left(-\frac{2\pi A d k_0}{\Lambda_{\mathrm{FL}}}\right) + \frac{\Lambda_{\mathrm{FL}}}{2}\sin\left[\frac{P\pi(n-1)}{Q}\right]J_v\left(-\frac{2\pi A d k_0}{\Lambda_{\mathrm{FL}}}\right), & \text{if } n \text{ is odd,} \\ \frac{\Lambda_{\mathrm{FL}}}{2}\sin\left(\frac{P\pi n}{Q}\right)J_v\left(-\frac{2\pi A d k_0}{\Lambda_{\mathrm{FL}}}\right) - \frac{\Lambda_{\mathrm{FL}}}{2}\cos\left(\frac{P\pi n}{Q}\right)E_v\left(-\frac{2\pi A d k_0}{\Lambda_{\mathrm{FL}}}\right), & \text{if } n \text{ is even.} \end{cases} \tag{S26}$$

Therefore,

$$H\left(\frac{\Lambda_{\mathrm{FBO}}}{2}\right) = \begin{cases} \text{i.} \quad \frac{\Lambda_{\mathrm{FL}}}{2}\left\{2E_v\left(-\frac{2\pi A d k_0}{\Lambda_{\mathrm{FL}}}\right) + 2\frac{\sin\left[\frac{(Q-2)P\pi}{2Q}\right]\sin\left(\frac{P\pi}{2}\right)}{\sin\left(\frac{P\pi}{Q}\right)}J_v\left(-\frac{2\pi A d k_0}{\Lambda_{\mathrm{FL}}}\right)\right\} & \text{when } Q \text{ is even,} \\ \text{ii.} \quad \frac{\Lambda_{\mathrm{FL}}}{2}\left\{E_v\left(-\frac{2\pi A d k_0}{\Lambda_{\mathrm{FL}}}\right) + 2\frac{\sin\left[\frac{(Q-1)P\pi}{2Q}\right]\sin\left[\frac{(Q+1)P\pi}{2Q}\right]}{\sin\left(\frac{P\pi}{Q}\right)}J_v\left(-\frac{2\pi A d k_0}{\Lambda_{\mathrm{FL}}}\right)\right\} & \text{when } Q \text{ is odd.} \end{cases} \tag{S27}$$

Equation (S27) indicates that the presence of harmonic modulation leads to a rescaling of FBO amplitude following the square of a linear combination of Anger function $J_v\left(\frac{2\pi A d k_0}{\Lambda_{\mathrm{FL}}}\right)$ and Weber function $E_v\left(\frac{2\pi A d k_0}{\Lambda_{\mathrm{FL}}}\right)$ with a fractional order v, which we call the fractional Floquet tunneling. The proposed fractional Floquet tunneling exhibits profound connection to the conventional tunneling that follows integral-order Bessel function. In absence of Bloch oscillations, Bloch oscillation period can be considered as infinite and $v = \Lambda_{\mathrm{FL}}/\Lambda_{\mathrm{BO}}$ approach zero and thus fractional Floquet tunneling degenerate into conventional tunneling that follows zero-order Bessel function for dynamic localization[4,5]. When the rational ratio of $\Lambda_{\mathrm{FL}}/\Lambda_{\mathrm{BO}}$ is integer, fractional Floquet tunneling degenerate into conventional tunneling that follows integer-order Bessel function as predicted in spreading[6,7].

In the main manuscript, we consider two examples of such Floquet tunneling, namely quasi-BOs (FBOs with $\Lambda_{\mathrm{BO}}/\Lambda_{\mathrm{FL}} = 3$) and SBOs-like oscillations (FBOs with $\Lambda_{\mathrm{BO}}/\Lambda_{\mathrm{FL}} = 4/3$). For quasi-BOs ($v = \Lambda_{\mathrm{FL}}/\Lambda_{\mathrm{BO}} = 1/3$), $H\left(\frac{\Lambda_{\mathrm{FBO}}}{2}\right) = 2\cos(\pi/3)E_v\left(-\frac{2\pi A d k_0}{\Lambda_{\mathrm{FL}}}\right) + 2\sin(\pi/3)J_v\left(-\frac{2\pi A d k_0}{\Lambda_{\mathrm{FL}}}\right)$ can be derived. For SBOs-like oscillations ($v = \Lambda_{\mathrm{FL}}/\Lambda_{\mathrm{BO}} = 3/4$), $H\left(\frac{\Lambda_{\mathrm{FBO}}}{2}\right) = 2\sqrt{2}J_v\left(\frac{2\pi A d k_0}{\Lambda_{\mathrm{FL}}}\right)$ can be derived.

To experimentally verify our prediction, we fabricated a set of photonic lattices with a fixed Bloch oscillation period $\Lambda_{\mathrm{BO}} = 30$ mm and a varied ratio A/Λ_{FL} ranging from 0 to 18×10^{-4} with an interval of 2×10^{-4}. With the single-site excitations, we captured the fluorescence image and extracted the corresponding variance $\sigma^2(z)$. To decrease the effect of noise, we took the average of $\sigma^2(z)$ ranging from $z = \Lambda_{\mathrm{FBO}}/2 - 250$ μm to $z = \Lambda_{\mathrm{FBO}}/2 + 250$ μm as the final result of $\sigma^2(\Lambda_{\mathrm{FBO}}/2)$. For the quasi-BO cases with the modulation period $\Lambda_{\mathrm{FL}} = 10$ mm, the modulation amplitude A is set as 0 to 18 μm with an interval of 2 μm. For the SBO-like cases with the modulation period $\Lambda_{\mathrm{FL}} = 22.5$ mm, the modulation amplitude A is set as 0 to 40.5 μm with an interval of 4.5 μm. Under the single-site excitations, the experimental observations of evolution patterns, respective simulations, and extracted variances $\sigma^2(z)$ for the scenarios are shown in Fig. S5 (quasi-BOs) and Fig. S6 (SBO-like oscillations).

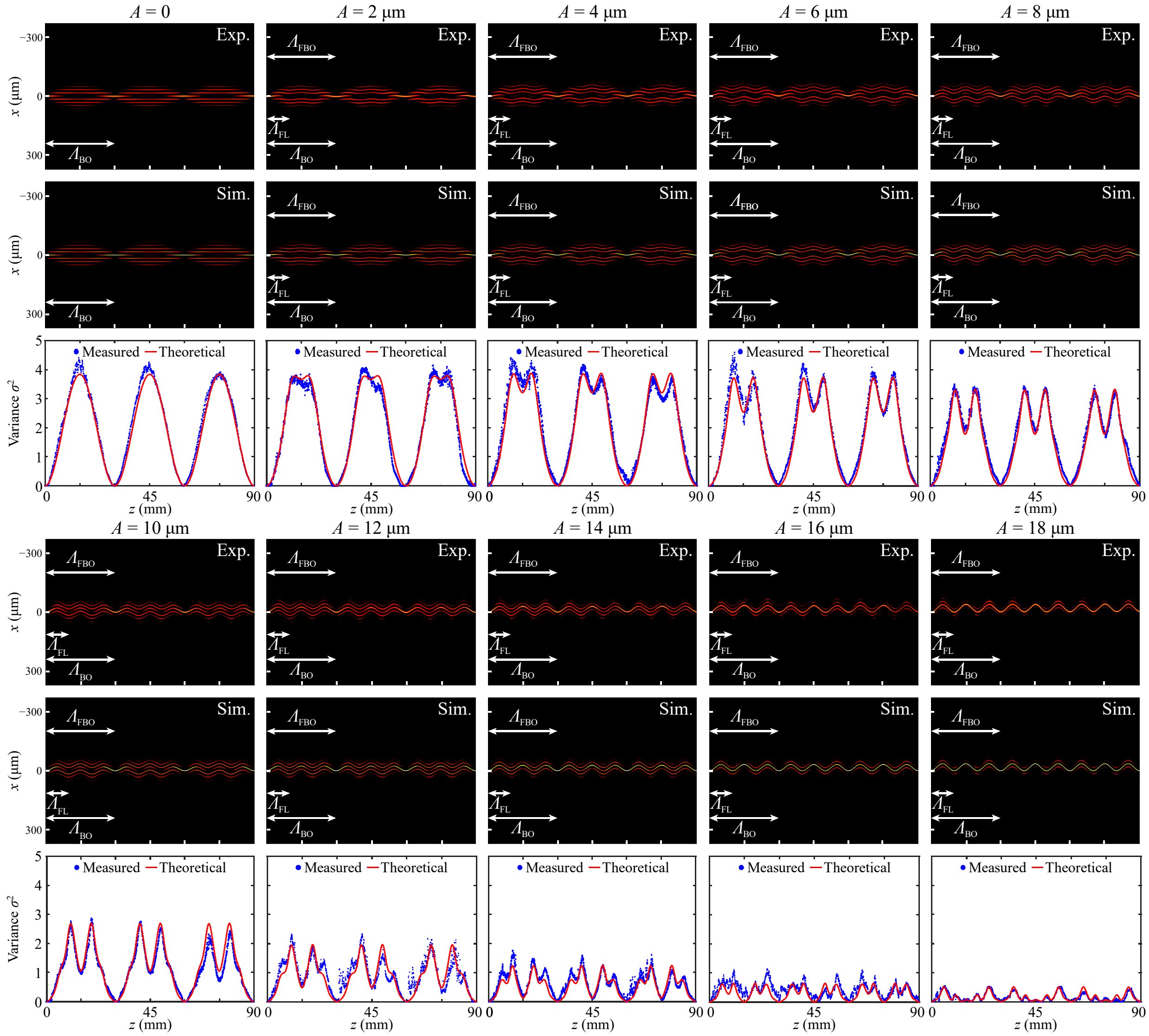

Fig. S5. Experimental visualization, simulation, and variance of the breathing modes for measuring the FBO amplitudes of quasi-BO cases. The modulation amplitude A starts from 0 to 18 μm with an interval of 2 μm, where experimental (Exp.) and simulated (Sim.) results are compared.

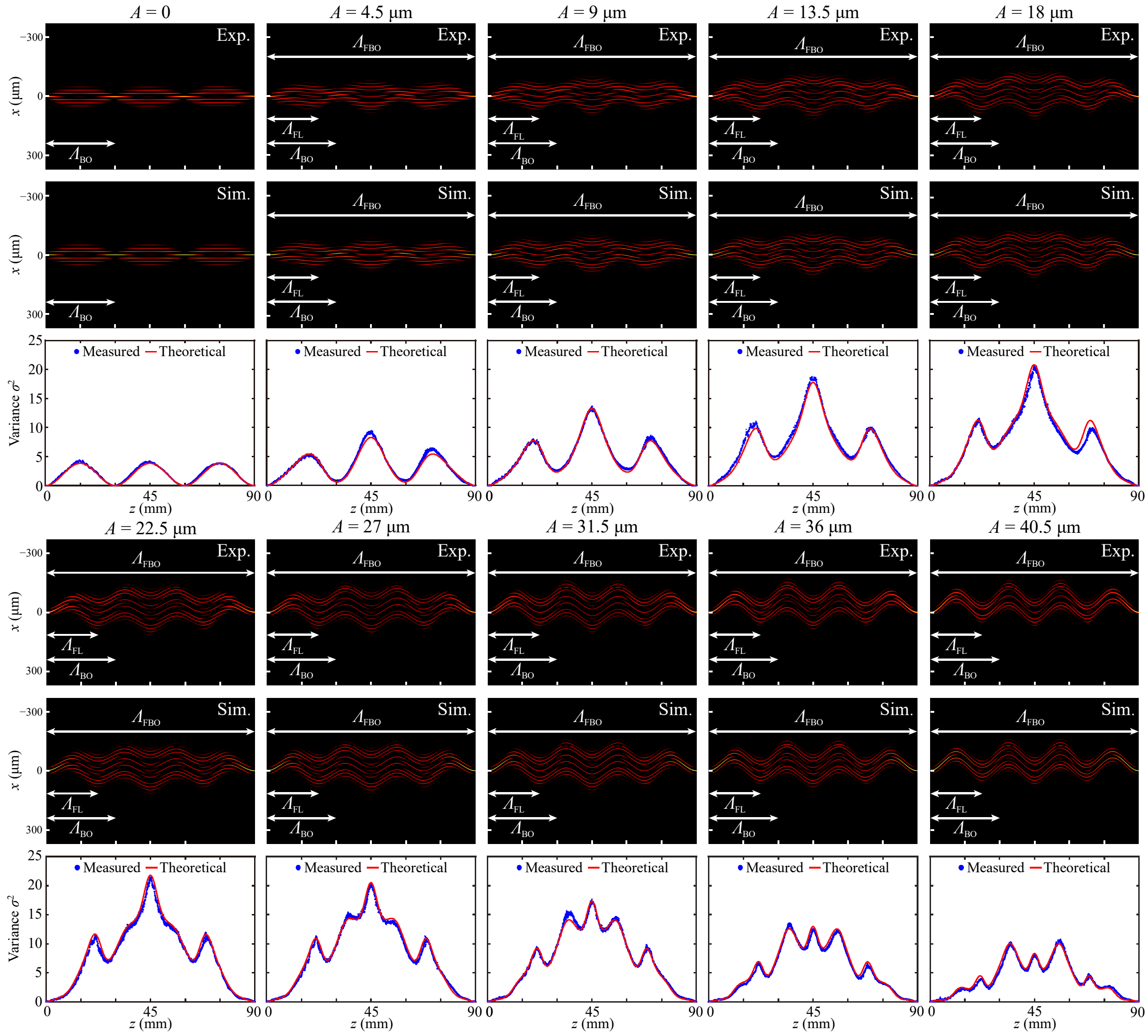

Fig. S6. Experimental visualization, simulation, and variance of the breathing modes for measuring the FBO amplitudes of SBO-like cases. The modulation amplitude A starts from 0 to 40.5 μm with an interval of 4.5 μm, where experimental (Exp.) and simulated (Sim.) results are compared.

References

1. Houston, W. V. Acceleration of electrons in a crystal lattice. *Phys. Rev.* **57**, 184–186 (1940).
2. Abbott, S. Understanding Analysis. 2nd edn. (New York: Springer, 2015).
3. Watson, G. N. *A Treatise on the Theory of Bessel Functions*. 1st edn. (Cambridge University Press, 1922).
4. Longhi, S., et al. Observation of dynamic localization in periodically curved waveguide arrays. *Phys. Rev. Lett.* **96**, 243901 (2006).
5. Tang, H., et al. Experimental quantum simulation of dynamic localization on curved photonic lattices. *Photonics Research* **10**, 1430–1439 (2022).
6. Sias, C., et al. Observation of photon-assisted tunneling in optical lattices. *Phys. Rev. Lett.* **100**, 040404 (2008).
7. Mukherjee, S., et al. Modulation-assisted tunneling in laser-fabricated photonic Wannier–Stark ladders. *New J. Phys.* **17**, 115002 (2015).
